# Cavity quantum electrodynamics control of quantum Hall stripes

**DOI:** 10.1038/s41567-026-03287-3

**Published:** 2026-05-15

**Authors:** Lorenzo Graziotto, Josefine Enkner, Sambuddha Chattopadhyay, Jonathan B. Curtis, Ethan Koskas, Christian Reichl, Werner Wegscheider, Giacomo Scalari, Eugene Demler, Jérôme Faist

**Affiliations:** 1 Institute for Quantum Electronics, ETH Zürich, Zurich, Switzerland; 2 Quantum Center, ETH Zürich, Zurich, Switzerland; 3 Institute for Theoretical Physics, ETH Zürich, Zurich, Switzerland; 4 Lyman Laboratory, Department of Physics, Harvard University, Cambridge, MA USA; 5 Laboratory for Solid State Physics, ETH Zürich, Zurich, Switzerland

**Keywords:** Quantum Hall, Quantum optics

## Abstract

Controlling quantum phases of materials with vacuum field fluctuations in engineered cavities is a topical method for the optical manipulation of emergent phenomena. Here we demonstrate cavity-induced anisotropies in the electronic transport of a high-mobility two-dimensional electron system in a strong magnetic field. In particular, we show the suppression of longitudinal resistivity well below the resistivity at zero magnetic field. These cavity-induced effects occur at ultralow temperatures when the magnetic field lies between quantized Hall plateaus. We interpret our results as arising from the stabilization of thermally disordered quantum Hall stripes. Therefore, our work presents a demonstration of the cavity quantum electrodynamics control of a correlated electronic phase.

## Abstract (web summary)

Vacuum fluctuations amplified by a cavity can control and modify quantum phases. Now, strong anisotropic transport is demonstrated by manipulating quantum Hall stripes in a two-dimensional electron gas.

## Main

The prospect of optically inducing correlated electronic phases of matter on demand in solid-state systems has materialized in the past decade due to rapid developments in the tailoring of electronic properties with strong electromagnetic fields^1^. A nascent complementary program is the control of quantum materials using the vacuum fields of engineered cavities, a mode of passive control in which effects can be achieved while the system is maintained at equilibrium^2–4^. Central to this approach is the notion that empty space contains vacuum fluctuations^5^ that give rise to canonical quantum electrodynamics effects such as the Casimir force^6^ or Lamb shift^7^. Shaping the electromagnetic environment by designing suitable resonators, thus, allows one to harness vacuum fields to influence material properties, an idea that has been explored theoretically in diverse contexts ranging from ferroelectricity^8–10^ and superconductivity^11–15^ to ferromagnetism^16^. Cavity effects, leveraging either thermal or vacuum fields, have recently been experimentally demonstrated by controlling the metal-to-insulator transition temperature in 1T-TaS_2_ (ref. ^17^) by coupling to graphene plasmons in a van der Waals heterostructure^18^ as well as by altering the transport properties of the integer and fractional quantum Hall effect in high-mobility two-dimensional electron systems (2DESs) at millikelvin temperatures^19,20^.

The quantum Hall system, which is realized by subjecting a 2DES to a perpendicular magnetic field *B* (ref. ^21^), represents an ideal playground for vacuum cavity control as the effective light–matter interaction length scales—set by the radius of the cyclotron orbit—are larger than in a typical solid by four orders of magnitude^22^. Additionally, the magnetic field quenches the kinetic energy of the system, leading to a number of energetically competing, correlated electronic phases defined by strong Coulomb interactions that are amenable to cavity control. Crucially, the correlated phases exhibited by the quantum Hall system depend on the number of occupied Landau levels (LLs)—which constitute its distinctive energy spectrum—quantified by the filling factor *ν* = *hn*_s_/*eB*, where *h* is the Planck constant, *e* is the electron charge and *n*_s_ indicates the two-dimensional electron density. Near *ν* = *N* + 1/2, with *N* > 4 being an integer, one such correlated electronic phase, known as quantum Hall stripes, arises.

Quantum Hall stripes are a form of electronically driven charge density wave order that manifest at ultralow temperatures, in half-filled high LLs. Electronic density modulation at the scale of the cyclotron radius (approximately 50 nm at *ν* = 8 + 1/2)—arising from the ring-like shape of the higher-LL wave functions^23–25^—becomes thermodynamically favourable below approximately 1 K. However, although energetic constraints select the wavelength of the density modulation, they do not discriminate between different orientations of the stripe order. Thus, in homogeneous and isotropic electron systems, such as those used in our study, thermal fluctuations scramble the orientation of the stripes, precluding their direct observation in magnetotransport measurements. Experimental signatures of stripes are observed when structural anisotropies aligned with a particular crystallographic direction of the heterostructure^26^, induced by strain^27^, or in-plane magnetic fields^28–30^ result in the alignment of the stripes on a macroscopic scale, giving rise to huge magnetotransport anisotropies measured near high half-integer filling factors^31,32^.

Here we demonstrate the cavity quantum electrodynamics control of quantum Hall stripes by means of the vacuum electromagnetic field of a slot antenna cavity, which was designed and engineered to realize a strongly anisotropic coupling with the 2DES, capable of steering the stripe order. Using our cavity, we induce not only large anisotropies in the longitudinal transport but also the nearly complete zeroing of the longitudinal resistivity far below its value in the absence of a magnetic field, away from quantizing magnetic field values. No other anisotropy-inducing mechanism^26–32^ has ever been demonstrated to achieve such an effect, which provides strong evidence of the capability of spatially structured cavity vacuum fluctuations to improve the transport in a correlated electronic phase.

**Fig. 1:**
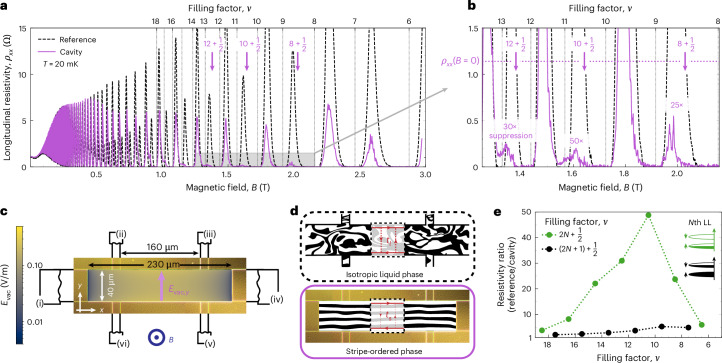
Cavity-induced stripe-ordered phase in the vacuum field of a slot antenna. **a**, Longitudinal resistivity *ρ*_*xx*_ as a function of perpendicular magnetic field *B* for a cavity-embedded HB (purple solid line) and a reference HB (black dashed line), measured along the $\widehat{{\bf{x}}}$ direction, as per the axes shown in **c**. The top axis reports the filling factor *ν* corresponding to *B* at a 2DES density of 4 × 10^11^ cm^−2^. **b**, Enlargement of the cavity-induced suppressed resistivity at filling factors of 12 + 1/2, 10 + 1/2 and 8 + 1/2 (grey rectangle in **a**), showing 30×, 50× and 25× reductions, respectively, compared with the resistivity measured in the reference HB. **c**, Optical microscopy picture of the slot antenna resonator, which consists of a 230 μm × 40 μm rectangular cutout from a metal plane (gold colour) evaporated on top of the HB. The gradient colour represents the vacuum electric field polarized in the $\widehat{{\bf{y}}}$ direction of the cavity fundamental mode, as obtained from finite-element simulations (Methods). The contact leads and continuation of the 40-μm-wide HB are visible as glowing lines below the metal. **d**, Cartoon of the two different phases of the 2DES without (top) and within (bottom) the cavity. In the middle of each HB, the basic process that scatters the edge states travelling in opposite directions into each other is sketched: in the stripe-ordered phase, the backscattering amplitude 1 − *t*_s_ is strongly reduced with respect to the one in the isotropic liquid phase 1 − *t*_l_. **e**, Ratio between the resistivity *ρ*_*xx*_ measured in the reference and in the cavity-embedded HBs, as a function of the filling factor. We observe a distinct behaviour when only the energetically lower spin-resolved LL is partially filled (green markers) or when the lower one is completely filled and the upper one is partially filled (black markers). The inset shows a sketch of the electronic occupation of the *N*th LL. Source data

**Extended Data Fig. 1:**
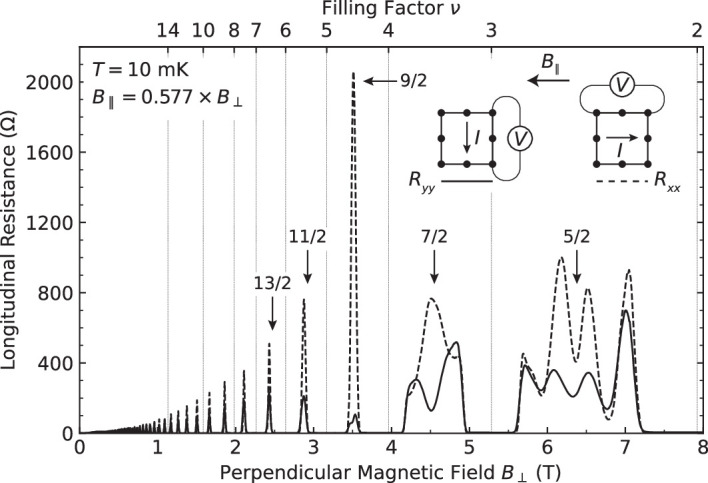
D170202B heterostructure. Longitudinal resistance as a function of perpendicular magnetic field *B*_⊥_ measured along the two orthogonal directions $\widehat{{\bf{x}}}$ (dashed line), parallel to the $(0\bar{1}\bar{1})$ GaAs crystallographic direction, and $\widehat{{\bf{y}}}$ (solid line), parallel to $(0\bar{1}1)$. The sample is tilted by 30^∘^, so to apply an in-plane magnetic field *B*_∥_ in the $\widehat{{\bf{x}}}$ direction, which varies linearly with *B*_⊥_, and it amounts to about 2 T at filling factor 9/2. The resistance *R*_*yy*_ has been multiplied by a factor of 1.5 to match *R*_*xx*_ in the low field regime, as conventionally done in the literature. The insets depict the two orthogonal contact schemes. Source data

In our experiment, we use a 2DES with mobility *μ* = 2.03 × 10^7^ cm^2^ V^−1^ s^−1^ and density *n*_s_ = 3.98 × 10^11^ cm^−2^ (measured at 1.3 K without illumination), realized in a high-quality epitaxially grown GaAs-based heterostructure (Methods), featuring excellent homogeneity and isotropy (Supplementary Information). We have verified that the application of an in-plane magnetic field results in the macroscopic alignment of stripes as it is regularly observed in the literature^28–30^ (Extended Data Fig. 1 and Supplementary Information), thereby confirming the microscopic existence of stripes in our 2DES. We fabricate a 40-μm-wide Hall bar (HB) embedded in the slot antenna cavity, and we measure its magnetotransport properties at high half-integer filling factors and ultralow temperatures, where vacuum fluctuations of the cavity field vastly dominate thermal fluctuations, as the photon population is below 10^−30^ at the lowest temperatures. In Fig. 1a,b, we report the main observation of our work: the 50-fold suppression of the longitudinal resistivity, measured along the $\widehat{{\bf{x}}}$ direction at filling factor *ν* = 10 + 1/2 and a temperature of 20 mK, in the cavity-embedded HB, compared with the reference HB. The latter was fabricated on the same chip—physically separated by a distance of about 2.5 mm—and measured in the same cool-down. The fact that the longitudinal resistivity is suppressed down to 0.2 Ω, well below its value at zero field of 1.15 Ω, demonstrates a suppression of backscattering away from quantized magnetic field values, and points to the stabilization by the cavity of a continuous stripe along the whole 160-μm distance between the voltage probes (Fig. 1c(ii),(iii)).

The substantial subwavelength confinement of the ground-state electromagnetic modes realized in the slot antenna cavity is a central ingredient in magnifying the amplitude of the vacuum fluctuations and, hence, their coupling to the 2DES, which is usually quantified by the normalized light–matter coupling *η*. Reference ^33^ showed that in this system, the effective cavity volume is $7\times 1{0}^{-4}{({\lambda }_{205{\rm{GHz}}}/2)}^{3}$ for the fundamental mode with a frequency of 205 GHz (ref. ^33^), and that *η* ≈ 20%: the light–matter coupled system is, thus, said to be in the ultrastrong-coupling regime^34^. The geometry of the cavity was chosen such as to provide a large number of modes (five; Methods) between 0.2 and 1.2 THz having the electric field polarized along the $\widehat{{\bf{y}}}$ direction (Fig. 1c), perpendicular to the edges of the resonator. As we show theoretically below, this favours the alignment of the stripes parallel to the edges—that is, with the wavevector of the charge density modulation parallel to the field polarization—rendering the transport easier along the $\widehat{{\bf{x}}}$ direction and harder along $\widehat{{\bf{y}}}$. A cartoon of the physical mechanism at play is depicted in Fig. 1d, contrasting the stripe-ordered phase of the 2DES inside the cavity with the isotropic liquid phase manifested in the absence of it.

**Extended Data Fig. 2:**
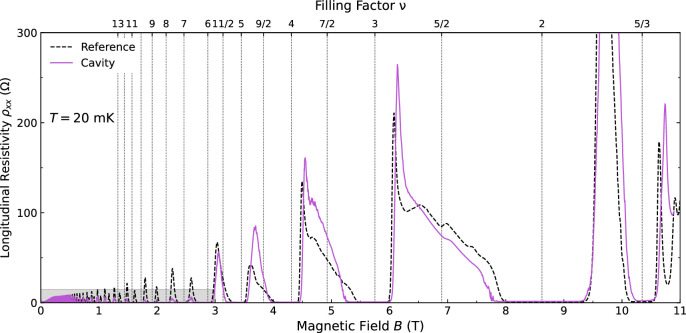
Sample D170202B-2. Longitudinal resistivity as a function of perpendicular magnetic field *B*, up to 11 T, for the reference (black dashed line) and cavity-embedded (purple solid line) HBs. The grey rectangle indicates the magnetic field region up to 3 T shown in Fig. 1a in the Main text. We notice in particular the absence of cavity-induced resistance suppression at filling factor *ν* = 9/2 and 11/2, and the well-quantized 5/3 fractional quantum Hall state in both reference and cavity-embedded HBs. Source data

To realize and maintain the stripe order over the whole length of 160 μm that separates the voltage probes, we have crafted our slot antenna endowing it with smooth cutout edges at a submicrometre scale. Without this care, the reduction below the zero-field value is unattainable (a similar but lower suppression is observed also in 2DESs having different densities hosted in different heterostructures, and in our hovering cavity experiment^20^; Supplementary Information). To confirm this statement, we also fabricated and investigated cavities having stepped or serrated edges by design (Supplementary Information). We notice that the reduced resistivity in the cavity sample is not merely due to sample-specific features such as the different disorder configurations in the cavity-embedded and reference HBs, as the zero-field resistivity is not modified by the presence of the cavity. The consequently unmodified zero-field mobility also attests that the direct lithographic fabrication of the cavity on top of the sample does not compromise the quality of the 2DES. In addition, as already assessed in our previous study^19^, the fragile fractional quantum Hall states are only weakly affected by the cavity (the 5/3 state is developed with a similar quantization quality in both reference and cavity-embedded HBs; Extended Data Fig. 2). We mention in passing that in ref. ^20^, we showed instead that fluctuations from a hovering cavity can even improve the fractional states. The observed suppression is robust: it is measured in both magnetic field directions, and for increasing and decreasing field sweeps (Supplementary Information). Furthermore, it is even observed in samples featuring a different cavity design, having lower fundamental mode frequencies (Supplementary Information).

As shown in Fig. 1e, we observe the resistivity suppression, although to a lesser extent, at all half-integer filling factors between 6 + 1/2 and 18 + 1/2, with a consistent distinction between *ν* = 2*N* + 1/2, with *N* ≥ 3 being an integer, and *ν* = (2*N* + 1) + 1/2, where it amounts to about an order of magnitude less. We connect the latter observation to a different occupation of the spin-resolved *N*th LL: at *ν* = 2*N* + 1/2, only one spin polarization is present, whereas at *ν* = (2*N* + 1) + 1/2, the energetically lower spin-resolved level is full, and electrons with the opposite spin partially fill the upper level. An analogous spin dependence was also observed in refs. ^31,32^, and, in ref. ^35^, a lower critical temperature of the stripe-ordered phase was computed for the *ν* = (2*N* + 1) + 1/2 case as opposed to *ν* = 2*N* + 1/2. Since the critical temperature is linked to the exchange energy magnitude, we also mention that a strong reduction in the effective *g*-factor due to cavity vacuum fields was reported in ref. ^20^ and also observed to a greater extent in our sample, starting from a lower value in the reference sample (Supplementary Information). Such a reduction is also linked to the shift in the peak position with respect to the reference ones^36^ (Fig. 1b). We also point out that the cavity-induced resistivity suppression above 18 + 1/2 (that is, below 1 T) is not indicative of stripe order, since the spin-split peaks merge as a result of the collapse of the exchange interaction, which is the primal drive for the appearance of stripes. Instead, such a reduction is linked to the cavity-induced amplitude modulation of the Shubnikov–de Haas oscillations, as already reported in ref. ^33^.

**Extended Data Fig. 3:**
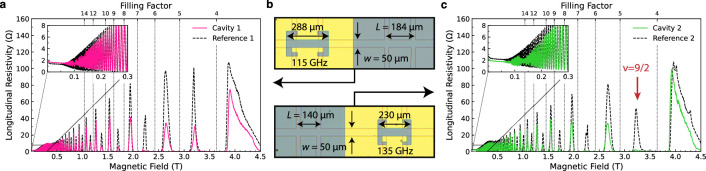
Sample D200923-Scs#1. (**a**) Longitudinal resistivity measured for a reference and a 115-GHz cavity-embedded HB, as a function of perpendicular magnetic field *B*. Around filling factor 6 + 1/2 = 13/2 we observe a ratio of about 21. (**b**) Optical microscope pictures of the two pairs of reference and cavity-embedded resonators. The first pair (top, data are presented in panel **a**) has distance between voltage probes of 184 *μ*m, while the second one (bottom, data are presented in panel **c**) has distance between voltage probes of 140 *μ*m. Both HB pairs have width 50 *μ*m. (**c**) Longitudinal resistivity measured for a reference and a 135-GHz cavity-embedded HB, as a function of *B*. For filling factors 6 + 1/2 = 13/2 and 4 + 1/2 = 9/2 we observe more than a 20-fold cavity suppression of the resistivity. The insets in panels **a** and **c** show an enlargement in the low-B region. Source data

We emphasize that the cavity-induced suppression is observed in a magnetic field range for which the cyclotron angular frequency *ω*_c_ = *eB*/m*, with m* = 0.067*m*_e_ being the electron effective mass in GaAs and *m*_e_ being the electron mass, which governs the optical response of the 2DES, is in a frequency range in which the cavity modes show anisotropy. Using a 2DES with reduced density, thus, allows to observe the same cavity-induced suppression at lower filling factors, including *ν* = 4 + 1/2 = 9/2, the value at which the stripe order is the most robust^31,32^ (Extended Data Fig. 3 and Supplementary Information).

**Fig. 2:**
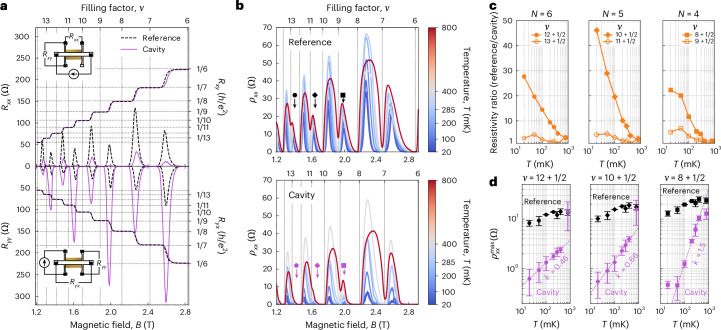
Magnetotransport in the stripe-ordered phase. **a**, Longitudinal and transverse resistances measured on a reference HB (black dashed lines) and on a cavity-embedded HB (purple solid lines), as a function of perpendicular magnetic field *B*, at 20-mK temperature. The left axes refer to the longitudinal resistances *R*_*xx*_ (top) and *R*_*yy*_ (bottom; with an inverted axis), whereas the right axes refer to the transverse resistances *R*_*xy*_ (top) and *R*_*yx*_ (bottom; with an inverted axis). The insets show the measurement schemes used to measure the different resistances. **b**, Longitudinal resistivity *ρ*_*xx*_ = *R*_*xx*_/4 as a function of *B* for the reference (top) and cavity-embedded (bottom) HBs, measured at different temperatures of the mixing-chamber plate (colours according to the colour bar on the right). **c**, Ratio between resistivity *ρ*_*xx*_ measured in the reference and cavity-embedded HBs as a function of temperature, for filling factors of 2*N* + 1/2 (full markers) and (2*N* + 1) + 1/2 (empty markers). The data are taken from the plots in **b**: circle, diamond and square markers refer to *N* = 6, 5 and 4, respectively, as indicated on top of the three subplots. **d**, Longitudinal resistivity maxima at half-integer filling factors as a function of temperature, in the log–log scale. Again, the data are taken from the plots in **b**: circles, diamonds and squares refer to filling factors of 12 + 1/2, 10 + 1/2 and 8 + 1/2, respectively, whereas black and purple colours refer to the reference and cavity sample, respectively. The resistivity maxima of the cavity sample follow a power-law behaviour as a function of temperature, ${\rho }_{xx}^{\max }\propto {T}^{k}$, with *k* indicated in the subplots. The error bars correspond to the standard deviation of the data measured in both magnetic field directions, and for increasing and decreasing *B*-field sweeps. Source data

We characterize the anisotropic magnetotransport in the stripe-ordered phase by comparing the longitudinal resistance *R*_*xx*_, measured along the $\widehat{{\bf{x}}}$ direction (Fig. 2a, top), with the resistance *R*_*yy*_, measured via the scheme depicted in the inset, which quantifies the transport in the orthogonal $\widehat{{\bf{y}}}$ direction (bottom). In complete agreement with our interpretation, we observe an increase in *R*_*yy*_ of more than a factor of 5 in the cavity-embedded HB at the same filling factors 8 + 1/2 and 10 + 1/2 for which the longitudinal resistance *R*_*xx*_ is suppressed—compared with the reference HB. As discussed in detail in Supplementary Information, the increase by a factor of 5 instead of 50 is due to the so-called non-local edge-state contribution to *R*_*yy*_ (ref. ^37^). As expected, the transverse resistance displays well-quantized plateaus^38^. The cavity and reference differ slightly only in the plateau-to-plateau transition, due to the minimal density difference in the two HBs—estimated to be about 1% by fitting the *R*_*xy*_ slope near the zero field.

We investigate the appearance of the stripe order through the temperature dependence of longitudinal resistivity. As shown in Fig. 2b,c, we observe the cavity-induced transport signatures only at ultralow temperatures: the resistivity at 800 mK is the same between the cavity-embedded and reference HBs. This represents a further indication that the role of the correlations induced by the cavity vacuum fluctuations is paramount, as the mere fabrication of a metallic plane near the 2DES is not expected to provide such precise signatures at millikelvin temperatures. We further notice the substantial difference in the temperature behaviour of the resistivity maxima at half-integer filling factors in the reference and cavity samples (Fig. 2d): although the former shows at most a twofold increase with increasing temperature, the latter climbs as a power law over an order of magnitude between 20 and 500 mK.

We remark that in the present work, we focus on correlated, low-temperature transport at high half-integer filling factors, where the 2DES behaves as an isotropic liquid in the absence of a cavity (Fig. 1b), differently from our previous works reported in refs. ^19,20^, where integer and odd-denominator fractional filling factors were investigated. Moreover, the cavity used there was a complementary split-ring resonator (evaporated on the sample in ref. ^19^ and hovering above it in ref. ^20^) with a lower resonance frequency, the 2DES mobility was about 10%–15% lower, and its carrier density was half the value used here.

**Fig. 3:**
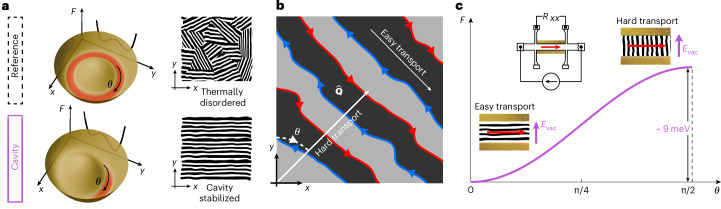
Orientational stabilization of fluctuating stripe order by the cavity. **a**, Free energy surface *F*(*θ*) as a function of the modulation direction of the stripe-ordered phase (displaced from the origin of the axes for visual clarity). Notice that a stripe modulation at angle *θ* means that the stripes are aligned along *θ* − 90°. In the reference case (top), the surface is rotationally invariant, so that the stripe order is formed with arbitrary orientation *θ*, and it thermally fluctuates (red shading of the *F* surface): domains of stripes with different orientations form in the reference sample, and no macroscopic orientation is present. In the presence of the cavity (bottom), the *F* surface possesses a clear minimum at *θ* = 0, which macroscopically aligns the stripes. **b**, Macroscopic stripe alignment, with modulation wavevector $\widehat{{\bf{Q}}}$, defines an axis along which transport is easy—orthogonal to $\widehat{{\bf{Q}}}$—and one along which transport is hard—parallel to $\widehat{{\bf{Q}}}$. **c**, Fixing the direction of the polarized vacuum electric field (*θ* = 0), the lowest free energy is obtained with a modulation parallel to it, thereby resulting in easy transport in the $\widehat{{\bf{x}}}$ direction, which is what is measured in our experiment.

We interpret our measurements by proposing that the anisotropic vacuum fluctuations of the electric field of the slot antenna cavity’s fundamental mode energetically favour the alignment of pre-existing but orientationally disordered local stripe order along the $\widehat{{\bf{x}}}$ axis (that is, charge order with density modulation with wavevector $\widehat{{\bf{Q}}}$ pointing along $\widehat{{\bf{y}}}$), as pictorially depicted in Fig. 3a. Such an interpretation provides a consistent picture for the main qualitative findings of our experiment. First, if stripes form along the $\widehat{{\bf{x}}}$ axis, then transport is easy along this direction, explaining the huge cavity suppression of the longitudinal resistivity *ρ*_*xx*_ (Fig. 3b). Second, stripes along the $\widehat{{\bf{x}}}$ axis imply that transport along the $\widehat{{\bf{y}}}$ axis is hard as it requires disorder-induced scattering processes across the stripes, justifying the increase in *R*_*yy*_ (Fig. 3b). Third, stripes give rise to anisotropic magnetotransport, as observed in our experiment, in the vicinity of high half-integer filling factors (for example, *ν* = 8 + 1/2, 10 + 1/2). Fourth, the temperature dependence of the cavity-induced signatures matches the regimes of stripe formation and alignment predicted by theory^24^ and observed in experiments^31,32^. In particular, the mean-field calculated critical temperature for the formation of the microscopic order is ${T}_{{\rm{c}}}^{{\rm{mf}}}\approx 0.02\,\hslash {\omega }_{{\rm{c}}}/{k}_{{\rm{B}}}$, with *ℏ* being the reduced Planck constant and *k*_B_ being the Boltzmann constant^24^. At a magnetic field of 2 T, corresponding to *ν* = 8 + 1/2, ${T}_{{\rm{c}}}^{{\rm{mf}}}\approx 800$ mK; therefore, microscopic stripe domains with arbitrary orientations will form below this temperature. In cavity-free samples the transport anisotropy has been observed up to 100–200 mK (refs. ^31,32^), which represents the critical temperature at which the macroscopic orientation is lost. The fact that we observe the cavity-induced suppression up to a much higher temperature of about half ${T}_{{\rm{c}}}^{{\rm{mf}}}$, thus, points to a much stronger aligning effect of the cavity, compared with the yet-unknown mechanism that aligns stripes in the cavity-free samples^31,32^. Finally, we underscore that magnetotransport in the sample is not intrinsically anisotropic, as discussed in Supplementary Information: the presumptive macroscopic stripe alignment is manifestly a cavity-induced effect. Again, the microscopic existence of stripe domains was separately confirmed by aligning them with an in-plane magnetic field, in agreement with the observations reported in the literature^28–30^, and the absence of macroscopic stripe order in the reference sample with zero in-plane field, at variance with the samples in which the stripes have been investigated in the literature, is attributed to the different details of the heterostructure growth, as discussed in Supplementary Information.

To conceptualize why the electromagnetic vacuum fluctuations of the cavity orient stripes along the $\widehat{{\bf{x}}}$ axis, we estimate the anisotropy in free energy arising from the interaction between a fixed orientation of stripes and the vacuum fluctuations of the electric field of the cavity. Physically, this can be interpreted as the change in the Casimir energy^39^ of the slot antenna cavity due to a particular orientation of electronic stripe order that anisotropically modifies the refractive index of the system. Within the Matsubara formalism^40^, we find that the free energy of a specific orientation of stripes is given by

1 $$F(\theta )=\frac{1}{\pi }\mathop{\sum }\limits_{a}{\int }_{0}^{\infty }{\rm{d}}\omega \,\omega {\sigma }_{aa}({\rm{i}}\omega ;\theta )\overline{\langle {{\bf{A}}}^{a}({\rm{i}}\omega ){{\bf{A}}}^{a}(-{\rm{i}}\omega )\rangle },$$

where the dynamical conductivity tensor—continued to imaginary frequencies—of stripes with modulation wavevector at an angle *θ* with respect to the cavity is given by *σ*_*ij*_(i*ω*; *θ*), and where the Matsubara frequency correlation function of the vector potential arising from the slot antenna is given by 〈**A**^*a*^(i*ω*)**A**^*b*^(−i*ω*)〉 = ∫d^2^**R**〈**A**^*a*^(**R**, i*ω*)**A**^*b*^(**R**, − i*ω*)〉 (Supplementary Information).

Our formula suggests that the stripe configuration with the lowest free energy is the one for which the hard axis of the conductivity—the axis for which the conductivity (resistivity) is smaller (larger)—is aligned with the axis of the resonator that experiences the largest amount of vacuum electromagnetic fluctuations. As the ultrastrongly coupled fundamental mode is polarized along $\widehat{{\bf{y}}}$, the cavity forces stripes to have their hard axis along the $\widehat{{\bf{y}}}$ direction, as consistent with our experimental observation. Within a canonical model of transport in the stripe phase^41^, we estimate that the anisotropy favouring alignment along the $\widehat{{\bf{x}}}$ axis amounts to a collective free energy difference between orthogonal (*θ* = 0 and *θ* = π/2) orientations of 9 meV, for the ~10^6^ electrons that constitute the highest partially filled LL, in qualitative agreement with previous estimates^42^ of the anisotropy required to reorient stripes (Fig. 3c). We remark that our theoretical treatment conceptualizes how vacuum fluctuations can alter correlated density-ordered states in high, half-filled LLs at ultralow temperatures, at variance with the vacuum-induced mechanisms proposed in refs. ^19,20^, dealing with phases close to quantized filling factors.

This interpretation underscores the potential that cavities hold in shaping equilibrium quantum fluctuations to manipulate correlated phases and situates our technique within the broader field of material control with light^1^. Crucially, our passive control scheme delicately selects the phase of the correlated stripe order and maintains its amplitude, in contrast with traditional laser-based techniques that melt the underlying order entirely^43^.

Cavity quantum electrodynamics control entails the intentional design of the electromagnetic environment to manipulate the emergent electronic properties of a quantum material. As has been proven successful in the broader optical control program^44^, cavities may be used to selectively stabilize fluctuating order in electronic systems. Our demonstration suggests to apply this approach to mesoscopic systems—particularly moiré materials^45^—which harbour many of the same characteristics that make quantum Hall a model setting for cavity control: crowded phase diagrams arising from the elevated importance of electronic interactions in the presence of quenched kinetic energy and large effective dipole sizes. Although magnetotransport anisotropy has predominantly been used to diagnose quantum Hall stripes, complementary approaches could be used, as transport is generally sensitive only to the phases present at the edges of the sample. Further evidence could be generated by examining the collective modes of the 2DES, exploring their plausible anisotropic dispersion. Previously, the collective mode spectrum of stripes has been mapped out using a combination of microwave driving with surface acoustic waves^46^, although scanning near-field optical microscopy techniques could also be applied^47^. Direct confirmation may come from real-space imaging: single-electron-transistor measurements^48^ have accessed the relevant 100-nm length scales at which stripes are expected to form. These techniques could also help resolve the possible coexistence of a stripe phase on the edges and a bubble phase in the bulk^49^.

## Methods

### Heterostructure details

The 2DES is hosted in a single rectangular 24-nm-wide GaAs/Al_0.25_Ga_0.75_As quantum well grown via molecular-beam epitaxy in the Laboratory for Solid State Physics at ETH Zürich (the heterostructure is labelled D170202B, and here we will refer to the processed chip as D170202B-2). The well is modulation doped with two Si δ-doping layers, with a 70-nm spacer between the doping layer and the edge of the barrier. Overall, the 2DES is located 130 nm below the surface of the heterostructure. At millikelvin temperatures from the Hall resistance slope, we obtain a density varying between 4.17 × 10^11^ and 4.36 × 10^11^ cm^−2^ across the sample, increasing over a length scale of about 3.3 mm. The isotropy of the bulk resistivity is discussed in Supplementary Section III; the macroscopic alignment of quantum Hall stripes with an in-plane magnetic field, in Supplementary Section IV; and the procedure used to account for density gradients in the estimate of the longitudinal resistivities from the HB resistance measurements, in Supplementary Section V.

### Sample fabrication

The samples are processed via standard photolithography and microfabrication techniques in a cleanroom environment. The procedure is carried out on a 9 × 10-mm^2^ chip cleaved from the molecular-beam-epitaxy-grown wafer, and it consists of the following steps:

1. Definition of the HBs and of the square patch using a positive resist (AZ1505) and high-resolution direct laser writing;
2. Etching of the mesa region using a diluted piranha solution (H_2_SO_4_:H_2_O_2_(30%):H_2_O at 1:8:60). The etch depth is measured to be 160 nm;
3. Definition of the contacts using negative image-reversal resist (AZ5214E) and high-resolution direct laser writing;
4. Evaporation of Ge/Au/Ge/Au/Ni/Au (26/53/26/53/40/50 nm) eutectic mixture, lift-off and annealing (500 °C for 300 s);
5. Definition of the resonator plane using negative image-reversal resist (AZ5214E) and high-resolution direct laser writing;
6. Evaporation of Ti/Au (10/200 nm) and lift-off.

### Measurement scheme

The transport measurements are performed with state-of-the-art techniques optimized for investigating the quantum Hall effect in 2DESs^50^. We cool the samples down to 12 mK with a Bluefors dilution refrigerator. The voltage is measured with commercial MFLI Zurich Instruments digital lock-in amplifiers, in conjunction with custom-made differential a.c. low-noise preamplifiers, which increase the signal by a factor of 1,000. We inject current symmetrically by applying an a.c.-modulated voltage of 2-V root-mean-square (r.m.s.) at the modulation frequency 13.333 Hz to a pair of 100-MΩ resistors in series with the sample (or equivalently applying 0.2 V to two 10-MΩ resistors), such that a 10-nA r.m.s. current circulates in the circuit, a low value chosen to limit electronic heating of the sample. To demodulate the lock-in input signal, we use a fourth-order low-pass filter with a time constant of 0.1 s. Before the contacts to the sample, low-pass 100-kHz filters are placed with the purpose of minimizing electric spikes or heating effects from the measurement setup.

### Finite-element simulations

**Extended Data Fig. 4:**
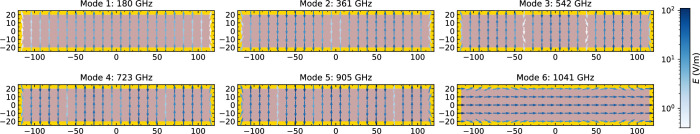
Finite-element simulations. Electric field profile for the six lowest frequency modes inside the slot antenna cavity. The yellow colour indicates the gold plane around the 230 × 40-*μ*m^2^ cutout. The arrows indicate the local polarization of the field, and the amplitude $E=\sqrt{{E}_{x}^{2}+{E}_{y}^{2}}$ is indicated according to the colour bar on the right.

To characterize the mode structure of the slot antenna cavity, finite-element simulations are performed using the commercial CST Studio Suite software. We model the system as a lossy gold plane of 0.2-μm thickness, with the 230 × 40-μm^2^ slot cutout filled with loss-free GaAs. The substrate is also GaAs and an effective layer thickness is used to reduce the computational cost. For the simulation reported in Fig. 1c, the 2DES is modelled using a gyrotropic material with the magnetic field oriented perpendicular to the surface. In this section, instead, we do not model the 2DES and we focus on characterizing higher-order modes, which are excited via a waveguide port (the frequency is slightly reduced with respect to the case in which the 2DES is present). In Extended Data Fig. 4, the electric field spatial profile of the six lowest-frequency modes is displayed. As reported in the main text, the five lowest-frequency modes are quasi-transverse-electric and have the electric field polarized along the $\widehat{{\bf{y}}}$ direction, whereas the sixth one (above 1 THz) is still quasi-transverse-electric but polarized along $\widehat{{\bf{x}}}$. This is consistent with the frequency ordering of modes in a rectangular waveguide with the same ratio between the long and short sides^51^. We interpret the classical electric field *E* obtained from the finite-element simulations as the r.m.s. fluctuations of the quantum electric field $\sqrt{\langle {E}^{2}\rangle }$, by performing a renormalization such that the energy contained in the mode (in the region bounded by the slot cutout and extended in the orthogonal direction up to the depth at which *E* decays by a factor 1/*e* with respect to its maximum) is equal to *hf*/2, where *h* is the Planck constant and *f* is the mode frequency. This procedure gives higher field fluctuations at higher frequencies—in the same way as it is found in free space—but at higher frequencies, the structure ceases to behave as a resonator.

## Online content

Any methods, additional references, Nature Portfolio reporting summaries, source data, extended data, supplementary information, acknowledgements, peer review information; details of author contributions and competing interests; and statements of data and code availability are available at 10.1038/s41567-026-03287-3.

## Supplementary information

Supplementary Information Supplementary Sections I–IX, Figs. 1–25, Tables 1 and 2, and Discussion.

## Source data

Source Data Fig. 1 Source data for the plots shown in Fig. 1.

Source Data Fig. 2 Source data for the plots shown in Fig. 2.

Source Data Extended Data Fig. 1 Source data for the plots shown in Extended Data Fig. 1.

Source Data Extended Data Fig. 2 Source data for the plots shown in Extended Data Fig. 2.

Source Data Extended Data Fig. 3 Source data for the plots shown in Extended Data Fig. 3.

## Data Availability

The data that support the findings of this study are available via the ETH Research Collection at 10.3929/ethz-b-000723680 (ref. ^52^). Source data are provided with this paper.

## Code availability

The codes that support the findings of this study are available via the ETH Research Collection at 10.3929/ethz-b-000723680 (ref. ^52^).
