# Supplementary material for: Cavity quantum electrodynamics control of quantum Hall stripes
Source: Nat Phys. 2026 May 15;22(7):1094–9. doi: 10.1038/s41567-026-03287-3 (PMC13423813; doi:10.1038/s41567-026-03287-3)
Supplement: Supplementary file 1 — Supplementary Sections I–IX, Figs. 1–25, Tables 1 and 2, and Discussion. [file 41567_2026_3287_MOESM1_ESM.pdf]

nature physics

Supplementary information https://doi.org/10.1038/s41567-026-03287-3

Cavity quantum electrodynamics control of quantum Hall stripes

In the format provided by the authors and unedited

TABLE OF CONTENTS

I. Picture of the sample and data summary 2

II. Data at higher magnetic fields 3

III. Characterization of the intrinsic material anisotropy 4

IV. Stripes alignment with in-plane magnetic field 13

V. Longitudinal resistivity in the presence of a density gradient 16

VI. Nonlocal resistance and edge states transport model 18

VII. Experimental reproducibility 21
- A. Reproducibility across different magnetic field sweeps 21
- B. Measurement on a rotated cavity-embedded Hall bar 22
- C. Role of the cavity edges 22
- D. Reproducibility across different samples 24
  - 1. Second sample processed on D170202B (D170202B-1) 24
  - 2. Sample processed on D170209B (D170209B-1-40B) 25
  - 3. Sample processed on D200923A (D200923-Scs#1) 26
  - 4. Sample processed on D151202B (D151202B) 28
  - 5. Sample processed on D151202B, investigated in Ref. [1] 29

VIII. Temperature study of the odd-integer filling factor minima 31

IX. Theoretical Interpretation 34
- A. Generic Formulation 34
- B. Stripe Anisotropy 35

References 36

I. PICTURE OF THE SAMPLE AND DATA SUMMARY

An optical microscope picture of the processed chip is shown in Fig. S1, where the relative orientation with respect to the GaAs substrate crystallographic axes is also reported. This is known from the orientation of the primary flat of the wafer, and it has been double-checked by looking at the orientation of known surface defects. In Table S1 it is summarized where the measurements of the different samples are reported in the Main text or in the Supplementary Materials.

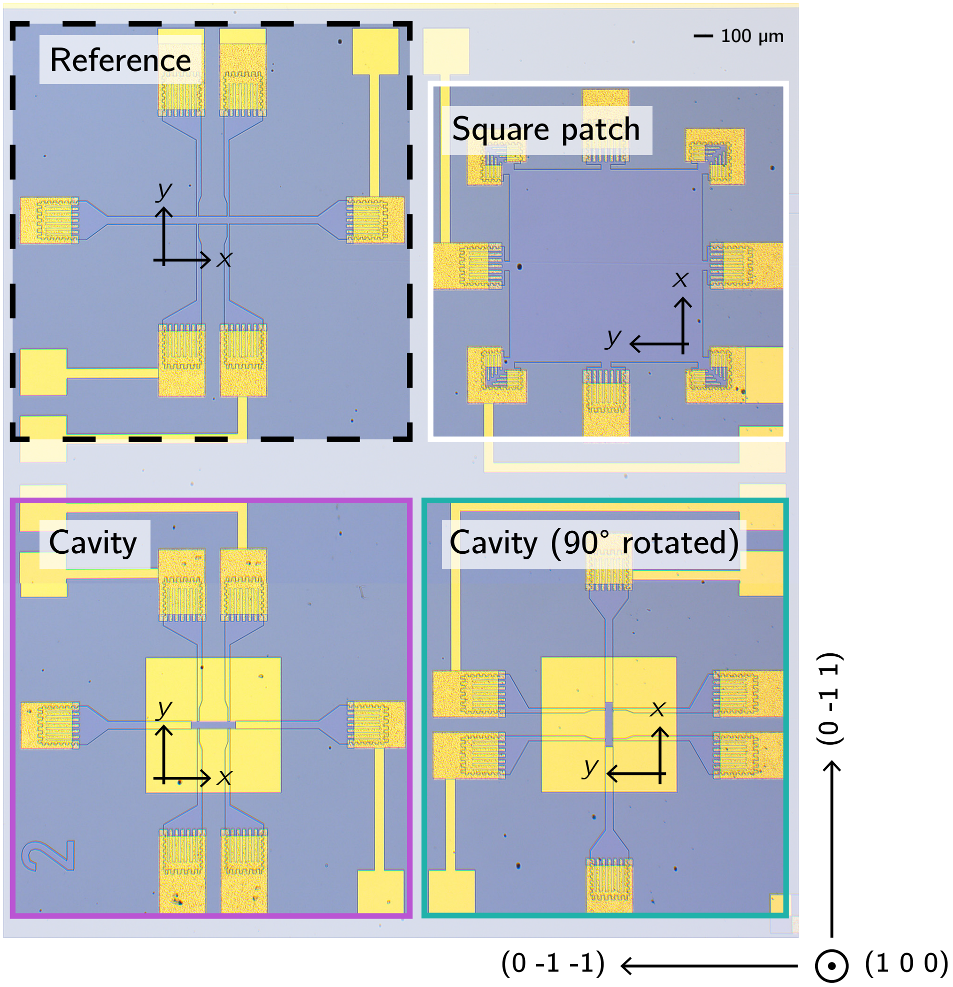

Figure S1. Optical microscope picture of the processed sample **D170202B-2**. The GaAs crystallographic directions are indicated below. Notice that the *Reference* and *Cavity* have the same orientation of the Cartesian axes, which is 90° rotated with respect to the *Square patch* and *Cavity (90° rotated)*.

Sample	Enlargement	Data
Reference		Figs. 1a,b, 2, S2, S13A,C, S14A, S15, S24, S25
Cavity	Fig. 1c	Figs. 1a,b, 2, S2, S13B,C, S14A, S15, S24, S25
Square patch	Fig. S3	Figs. S4, S5, S6A, S7, S8, S9
Cavity (90° rotated)		S16 (for **D170202B-1** only)

Table S1. Summary of the figures presenting the data from **D170202B-2**, referring to the samples shown in Fig. S1. The *Cavity (90° rotated)* is measured only in **D170202B-1** (which is a processed copy of **D170202B-2**).

II. DATA AT HIGHER MAGNETIC FIELDS

In Figure S2 (which is the same as Extended Data Fig. 2) we report the longitudinal resistivity measured in the reference and cavity sample for a larger range of magnetic fields with respect to the one discussed in the Main text. We observe how above 3 T the impact of the cavity presence on the magnetotransport is much less evident, specifically we do not observe the cavity-induced resistance suppression at filling factor $\nu = 9/2$ and $11/2$, which are the ones at which the stripe phase is the most robust [2, 3]. As discussed in the Main text, we attribute the absence of suppression at these filling factors to the fact that the corresponding cyclotron frequency is outside the frequency range in which the cavity modes show anisotropy. In Sec. VII D 3, we report measurements on a lower density sample, such that the cyclotron frequency at $\nu = 9/2$ lies within the frequency range in which the cavity orients the stripes, and where indeed we observe a cavity-induced resistance suppression at $\nu = 9/2$. The lack of anisotropy at $\nu = 7/2$ and below is instead consistent with all experiments reported so far in the literature.

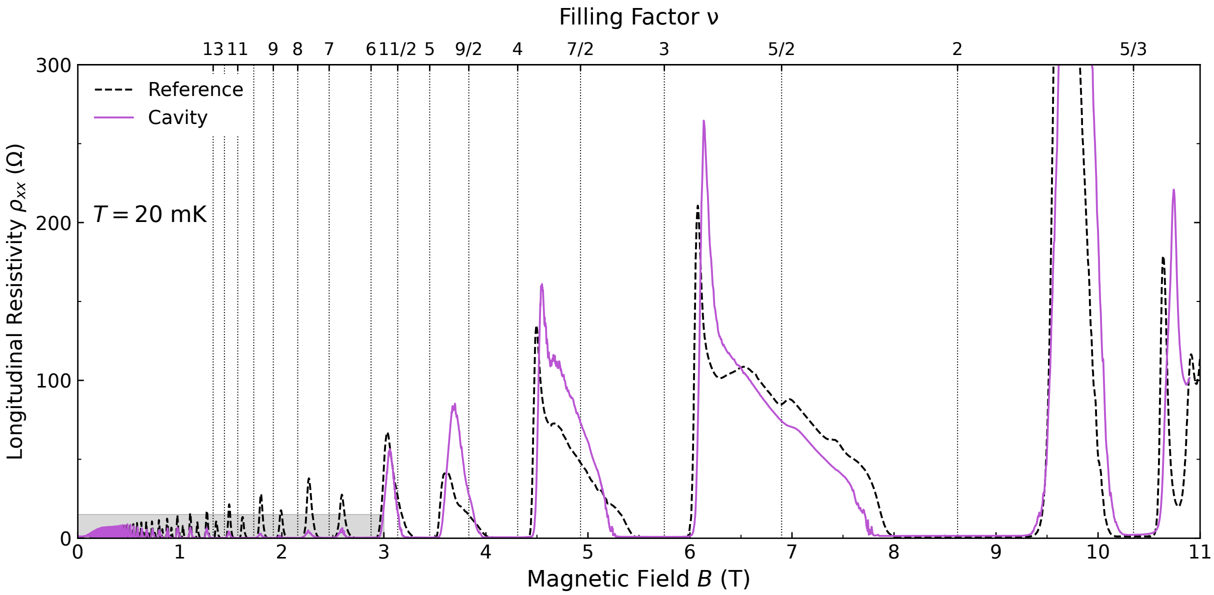

Figure S2. Sample **D170202B-2**—Longitudinal resistivity as a function of magnetic field, up to 11 T, for the reference (black dashed line) and cavity-embedded (purple solid line) HBs. The grey rectangle indicates the magnetic field region up to 3 T shown in Fig. 1A in the Main text. We notice in particular the absence of cavity-induced resistance suppression at filling factor $\nu = 9/2$ and $11/2$.

III. CHARACTERIZATION OF THE INTRINSIC MATERIAL ANISOTROPY

In this section we present and discuss transport measurements performed on a square patch with 1-mm side length, which is physically located on the same chip where the reference and the cavity-embedded Hall bars discussed in the Main text are (see Fig. S1). In this way, the square patch is fabricated concurrently with the Hall bar samples, it comprises a 2DES hosted in exactly the same heterostructure, and it is measured in the same cool-down run with the same measurement scheme. In the following, we show how the intrinsic material anisotropy can account for at most a factor of 1.18 difference in the longitudinal conductivities ρ_{xx} and ρ_{yy} along the two crystallographic directions, two to three orders of magnitude less than the cavity-induced anisotropy we report in the Main text.

a. Sample measurement — In Figure S3 we display an optical microscope picture of the square patch, along with the labeling of the contacts which we refer to in the following measurement plots. Transport is investigated via four-point resistance measurements: a current $I = 10\,\mathrm{nA}$ root-mean-square (rms) is symmetrically injected at contacts i, j (by applying $0.2\,\mathrm{V}$ rms on two $10\,\mathrm{M\Omega}$ resistors in front of the contacts, sinusoidally modulated at the lock-in $13.333\,\mathrm{Hz}$ oscillator frequency), and

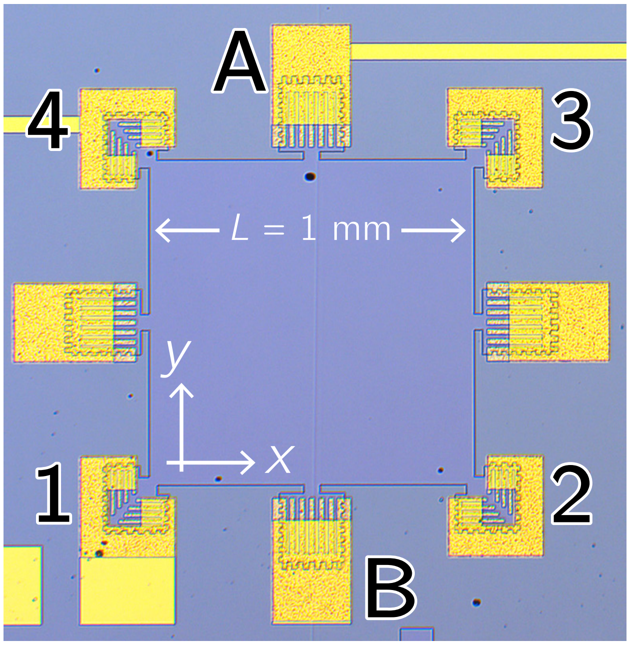

Figure S3. Optical microscope picture of the 1 mm-side square patch located on the same chip as the Hall bar samples discussed in the Main text. The numbers and letters label the Ohmic contacts nearby (the grainy yellow color comes from the metal annealing process), and we will refer to them in the following discussion. Notice the orientation of the Cartesian axes, which is rotated by 90° with respect to the one employed for the reference and cavity-embedded Hall bars (see Fig. S1).

the voltage difference is measured between contacts k, l. Dividing the latter by I, we obtain the resistance $R_{ij,kl}$.

In Figure S4 we report the longitudinal and transverse (Hall) resistance as a function of magnetic flux density (magnetic field) B, measured with different current injection and measurement contact pairs, labeled with the notation introduced above and marked for clarity in the insets. Although at some magnetic field values the longitudinal resistance traces differ almost by an order of magnitude, the difference is almost entirely explained by the presence of a density gradient through the sample, and leads to an almost entirely isotropic resistivity tensor.

b. Model for extracting the resistivity — To understand the origin of such a difference we elaborate on the model introduced in Ref. [4] to describe current flow in a 2DES with a gradient in the electronic density, assuming, at variance with the treatment therein, different longitudinal resistivities ρ_{xx}, ρ_{yy} along the two Cartesian axes. Our model allows us to extract both resistivities employing only the four longitudinal resistance traces shown in Fig. S4, without any additional adjustable parameter.

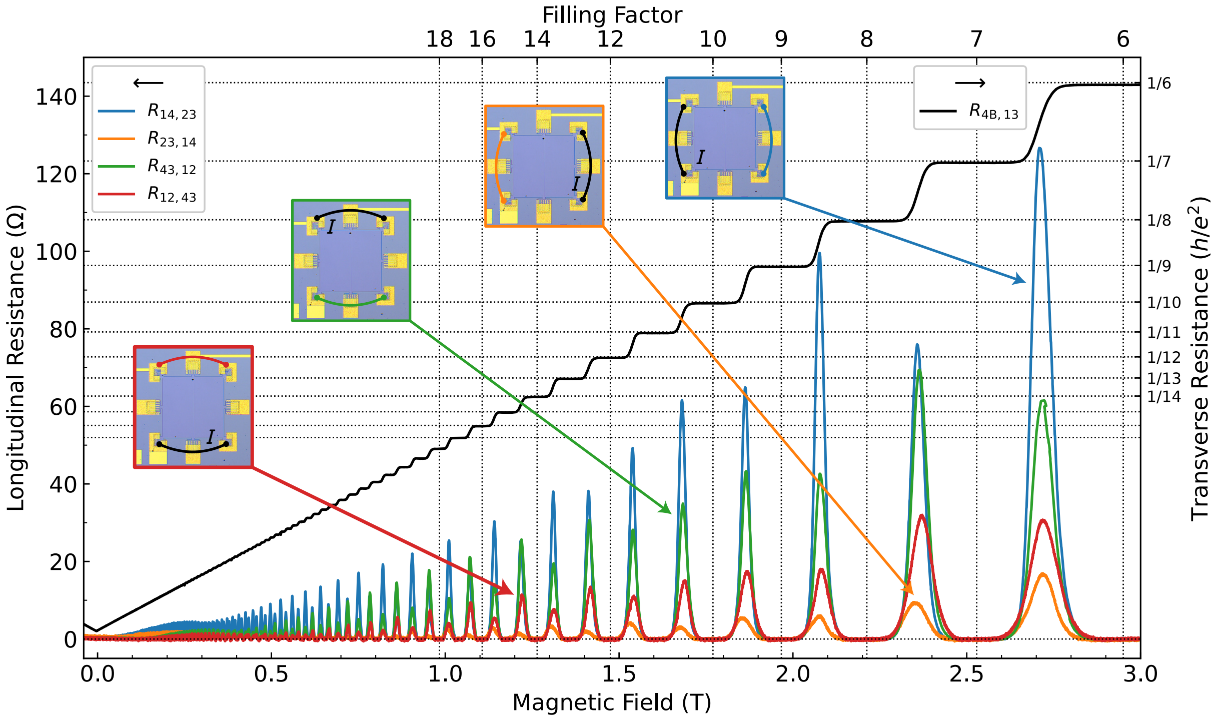

Figure S4. Longitudinal (left axis) and transverse (right axis) resistance of the square patch, as a function of magnetic field, for different current injection and measurement contact pairs, as depicted in the insets. The labeling $R_{ij,kl}$ in the legends employs the same notation introduced in Fig. S3. The top axis shows the filling factor corresponding to the magnetic field shown on the bottom axis.

The local electric field $\mathbf{E}(\mathbf{r})$ in the two-dimensional sample is given by

$$\mathbf{E}(\mathbf{r}) = \begin{pmatrix} \rho_{xx} & \rho_{xy} \\ -\rho_{xy} & \rho_{yy} \end{pmatrix} \mathbf{j}(\mathbf{r}), \tag{1}$$

where $\mathbf{j}(\mathbf{r})$ is the local current density, and the components of the resistivity tensor ρ_{ij} are also functions of position, due to inhomogeneities of the electron density in the sample. In the steady-state the following equations must hold

$$\boldsymbol{\nabla} \cdot \mathbf{j} = 0 \tag{2}$$

$$\boldsymbol{\nabla} \times \mathbf{E} = 0, \tag{3}$$

and we can ensure Eq. 2 by defining a stream function ψ via

$$\mathbf{j} = \begin{pmatrix} -\partial_y \psi \\ \partial_x \psi \end{pmatrix}. \tag{4}$$

Substituting Eq. 1 into Eq. 3, the equation for ψ is

$$\left(\rho_{yy}\partial^2_{xx}\psi + \rho_{xx}\partial^2_{yy}\psi\right) + \left(\partial_x\rho_{yy}\partial_x\psi + \partial_y\rho_{xx}\partial_y\psi\right) + \left(\partial_x\rho_{xy}\partial_y\psi - \partial_y\rho_{xy}\partial_x\psi\right) = 0, \tag{5}$$

which, together with the appropriate boundary conditions related to the current injection contacts, can be solved to yield the current distribution, and by integration the voltage drop between any pair of measurement contacts.

For magnetic field values in which the 2DES has a density close to a compressible state, and such that $|\rho_{xy}| \gg \rho_{xx}, \rho_{yy}$ (as it is the case for $B > 1\,\mathrm{T}$), we can assume that ρ_{xx}, ρ_{yy} are independent of position, while ρ_{xy} has a constant gradient $\boldsymbol{\nabla}\rho_{xy}$ in an arbitrary direction. This gradient reflects the density inhomogeneities present in the sample. Thus, Eq. 5 simplifies to

$$\left(\rho_{yy}\partial^2_{xx}\psi + \rho_{xx}\partial^2_{yy}\psi\right) + \left(\partial_x\rho_{xy}\partial_y\psi - \partial_y\rho_{xy}\partial_x\psi\right) = 0. \tag{6}$$

Without loss of generality, we can assume that current is injected and extracted from contacts 4 and 3, and that the voltage difference is measured between contacts 1 and 2, following the labeling of Fig. S3. The four configurations employed to measure the longitudinal resistances in Fig. S4 can be retrieved via a suitable rotation of Cartesian axes. According to the position of the injection contacts, the boundary conditions are

$$\psi(x, y = 0) = \psi(x = 0, y) = \psi(x = L, y) = 0 \tag{7}$$

$$\psi(x, y = L) = -I, \tag{8}$$

and we introduce the following length scales to quantify the carrier density variation across the sample

$$\ell_x \equiv \rho_{xx}\left(\partial_x\rho_{xy}\right)^{-1}, \tag{9}$$

$$\ell_y \equiv \rho_{yy}\left(\partial_y\rho_{xy}\right)^{-1}. \tag{10}$$

Using the separation of variables method, the solution to Eq. 6 is

$$\psi = -I\sum_{m=0}^{\infty}\frac{2m\pi\left(1-(-1)^m e^{-L/2\ell_y}\right)}{\left(\frac{L}{2\ell_y}\right)^2 + m^2\pi^2}\exp\left(\frac{x}{2\ell_y} - \frac{(y-w)}{2\ell_x}\right)\frac{\sinh\left(\frac{y}{2}\sqrt{\left(\frac{1}{\ell_x}\right)^2 + 4\lambda_m}\right)}{\sinh\left(\frac{L}{2}\sqrt{\left(\frac{1}{\ell_x}\right)^2 + 4\lambda_m}\right)}\sin\left(\frac{m\pi x}{L}\right), \tag{11}$$

where

$$\lambda_m = \frac{\rho_{yy}}{\rho_{xx}}\left(\frac{m^2\pi^2}{L^2} + \left(\frac{1}{2\ell_y}\right)^2\right). \tag{12}$$

We have numerically checked that for the length scales relevant in the experiment ($L = 1000\,\mu\mathrm{m}$, $\ell_x, \ell_y \gtrsim 100\,\mu\mathrm{m}$) we can retain only the term $m = 1$ in the sum without introducing a significant error. The voltage difference between contacts 1 and 2 (that is the voltage drop on the opposite side of the one where the current is injected) is given by

$$V_{12} = \int_0^L dx\, E(x, y = 0) = \rho_{xx} \int_0^L dx\, j_x(y = 0) = -\rho_{xx} \int_0^L dx\, \partial_y \psi|_{y=0}, \tag{13}$$

and the resistance is $R_{12} = V_{12}/I$. We can now adapt the solution to the different injection and measurement contact pairs, obtaining e.g.

$$R_{14,23} = \rho_{yy} \frac{\frac{1}{2}\sqrt{\frac{1}{\ell_y^2} + \left(\frac{1}{\ell_x^2} + \frac{4\pi^2}{L^2}\right)\frac{\rho_{xx}}{\rho_{yy}}}}{\sinh\left(\frac{L}{2}\sqrt{\frac{1}{\ell_y^2} + \left(\frac{1}{\ell_x^2} + \frac{4\pi^2}{L^2}\right)\frac{\rho_{xx}}{\rho_{yy}}}\right)} \frac{2\pi^2 L}{\left(\left(\frac{L}{2\ell_x}\right)^2 + \pi^2\right)^2} \left(1 + e^{L/2\ell_x}\right)\left(1 + e^{-L/2\ell_x}\right) e^{L/2\ell_y}, \tag{14}$$

where $\ell_x, \ell_y, \rho_{xx}, \rho_{yy}$ refer to the Cartesian system defined in Fig. S3. All other resistances can be obtained by either changing ℓ_x to ℓ_y, or ρ_{xx} to ρ_{yy}, and vice-versa. From the ratio between the resistances measured on two opposite sides (interchanging injection and measurement contact pairs) we immediately obtain the density inhomogeneity length scales

$$\ell_x = \frac{L}{\log(R_{43,12}/R_{12,43})}, \tag{15}$$

$$\ell_y = \frac{L}{\log(R_{14,23}/R_{23,14})}, \tag{16}$$

which can then be substituted back in Eq. 14 and the analogous ones, to obtain ρ_{xx} and ρ_{yy}. Notice that the four equations for the four longitudinal resistance traces contain only the four unknowns $\ell_x, \ell_y, \rho_{xx}, \rho_{yy}$, so we do not need to know beforehand the density inhomogeneity magnitude, apart from assuming that its gradient is constant. We remind that the transverse resistivity ρ_{xy} is instead simply equal to the transverse resistance $R_{4\mathrm{B},13}$. In Fig. S5 we report both the longitudinal and transverse resistivities of the square patch, as obtained with the procedure described above. Notice that the 2DES displays at most an 18% anisotropy: for filling factor $7 + 1/2$, $\rho_{xx} \simeq 1.18\rho_{yy}$. The values of ℓ_x, ℓ_y obtained at the half-integer filling factors range between 1 mm – 1.5 mm and 0.3 mm – 0.5 mm, respectively—that is, the density gradient is stronger in the y direction, and the density decreases along the y direction since $\ell_y > 0$—and they are consistent with the density gradient estimated by measuring the Hall resistance on different Hall bars located at different points on the chip. We remark once more that the model we have employed here completely disregards nonlocal effects due to the presence of edge states, and disequilibrium between edge and bulk transport channels.

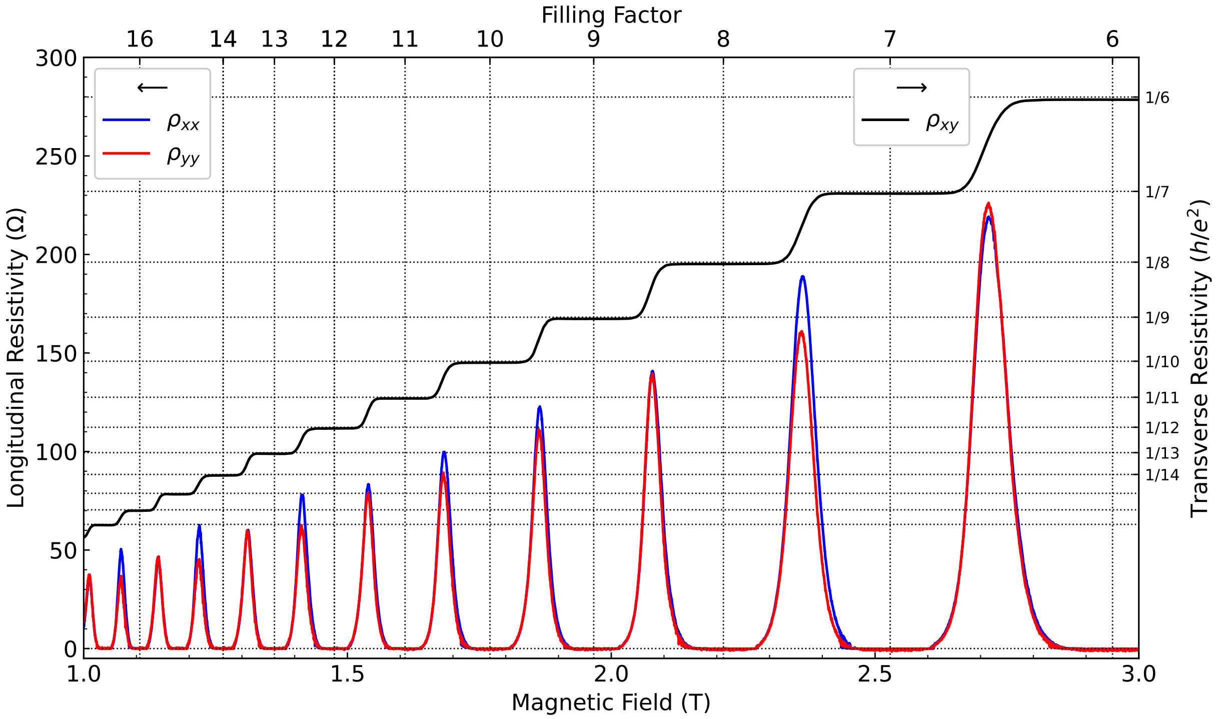

Figure S5. Longitudinal (left axis) and transverse (right axis) resistivity of the square patch, as a function of magnetic field, between 1 and 3 T. The longitudinal resistivities have been obtained from the longitudinal resistance traces shown in Fig. S4 via the analytical procedure described in the text, while the transverse resistivity coincides with the transverse resistance. The blue and red curves refer to ρ_{xx} and ρ_{yy}, respectively.

We have also experimentally verified that Onsager's relations [5] hold upon the simultaneous interchange of current and measurement contacts and magnetic field reversal, i.e.

$$R_{ij,kl}(+B) = R_{kl,ij}(-B), \tag{17}$$

such that measurements at negative magnetic field do not add additional information (see Fig. S6A).

c. On the lack of anisotropy — We can now comment on the almost entirely isotropic resistivity of our material at variance with what was observed in Ref. [2, 3]. First of all, due to simple geometrical reasons related to the current distribution inside the sample (see Fig. S6B), even a small anisotropy ratio is strongly enhanced by employing a square patch [6], and special care should be applied when extracting the longitudinal resistivities from the resistance measurements, following the procedure we explained above. According to Ref. [6], indeed, an anisotropy ratio of $\rho_{yy}/\rho_{xx} \approx 7$ is sufficient to produce the ratio $R_{yy}/R_{xx} \approx 60$ observed by Lilly *et al.* [2] in a square sample. Moreover, the square samples which have shown stripe anisotropy are usually obtained by cleaving a 4×4 mm^2 chip and annealing contacts on its sides, while in the present work the samples are

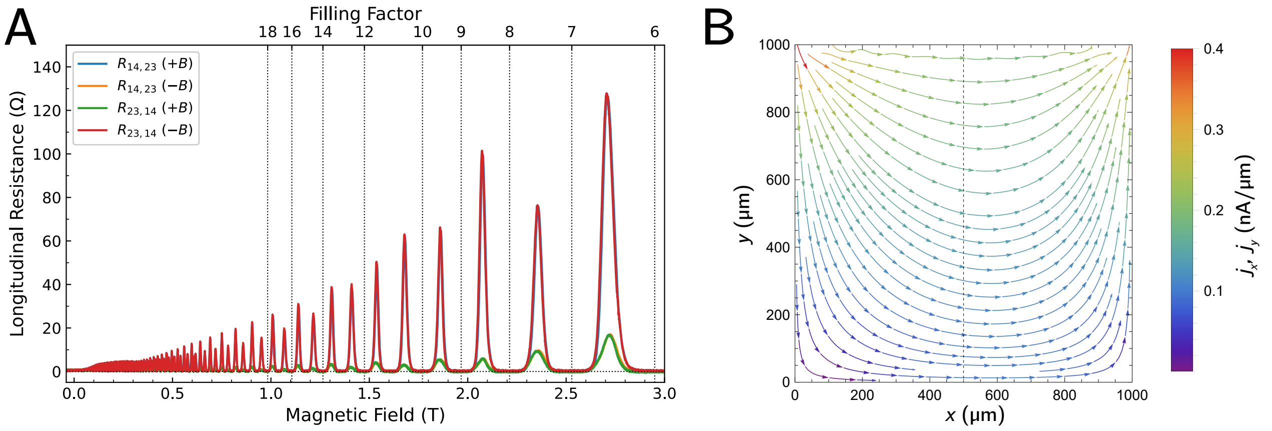

Figure S6. (**A**) Longitudinal resistance for two pairs of current and voltage probes (following the labeling of Fig. S3) for positive and negative magnetic field direction, as indicated in the legend. Notice how Onsager's relations hold. (**B**) Current density distribution inside the square sample (when current is injected through contacts 4, 3 as labelled in Fig. S3), as calculated with the model described in the text, and employing the values of $\ell_x \approx 1.4\,\mathrm{mm}, \ell_y \approx 0.5\,\mathrm{mm}, \rho_{xx} = \rho_{yy} \approx 220\,\Omega$ obtained experimentally at filling factor $6 + 1/2$, where the sample is entirely isotropic. Notice the strong decay of the current density magnitude towards the lower end of the sample. The asymmetric profile noticeable when looking along $x = 500\,\mu\mathrm{m}$ (dashed line) is due to the carrier density gradient.

fabricated by lithography.

Secondly, the mechanism responsible for macroscopically aligning the preexisting microscopic stripe domains is still debated in the literature, as thoroughly surveyed in Ref. [7]. Therein, the magnetotransport anisotropy was investigated in quantum wells having different widths and distance from the surface, so it is worth mentioning that the quantum well that hosts the 2DES in our sample is narrower (24 nm) than the ones reported there and in general in the literature about stripes (about 30 nm), and it sits at a lower distance from the surface (130 nm, see Methods). These details should not impact the energetics of microscopic stripe formation (which are determined by the relative strength of the Hartree and exchange energies [8]), but only their macroscopic alignment. Moreover, for 2DES densities similar to the one we employ ($4 \times 10^{11}\,\mathrm{cm}^{-2}$) an orientation of stripes along the $(1\bar{1}0)$ direction, contrary to the usual (110) direction observed in almost all other samples, was reported in Refs. [9, 10]. In Ref. [11] it was instead observed for the same density the conventional (110) alignment, establishing that high density alone is not a decisive factor in determining the unusual orientation.

Factors that affect the macroscopic alignment of stripes in the bare material could be related to the different growth conditions employed for this heterostructure, including growth temperatures,

rates, aluminum content at the interface, growth interruptions at the interfaces and As-flux. These parameters are typically not reported in the literature, but have been compared to the one in use at ETH Zürich through private communications with the MBE labs in Princeton and Purdue University.

To clearly demonstrate the existence of stripes in our heterostructure material, we align them macroscopically by applying an in-plane magnetic field, whose impact on the stripes orientation is known in the literature, and we present the results of such experiment in Sec. IV.

d. Conductivity and the semicircle rule — Inverting the resistivity tensor one obtains the conductivity tensor

$$\begin{pmatrix} \sigma_{xx} & -\sigma_{xy} \\ \sigma_{xy} & \sigma_{yy} \end{pmatrix} = \begin{pmatrix} \rho_{xx} & \rho_{xy} \\ -\rho_{xy} & \rho_{yy} \end{pmatrix}^{-1} = \frac{1}{\rho_{xx}\rho_{yy} + \rho_{xy}^2} \begin{pmatrix} \rho_{yy} & -\rho_{xy} \\ \rho_{xy} & \rho_{xx} \end{pmatrix}, \tag{18}$$

and we can thus verify that the sample fulfills the semicircle relation [12] adapted to the case of a generally anisotropic tensor [13]:

$$\sigma_{xx}\sigma_{yy} + \left(\sigma_{xy} - (N + 1/2)e^2/h\right)^2 = (e^2/2h)^2, \tag{19}$$

where N is a non-negative integer. In Fig. S7 we show $\sqrt{\sigma_{xx}\sigma_{yy}}$ as a function of σ_{xy}, which closely follows the semicircle relation (shown with the dashed line). Lack of perfect agreement comes from the finite size of the 1 mm × 1 mm square patch. Size effects are also manifest when comparing (Fig. S8) the product of the longitudinal resistivities, which according to Ref. [14] should be given, at half-integer filling factors ν and even in the presence of anisotropy, by

$$\rho_{xx}\rho_{yy} = \left(\frac{h}{e^2}\right)^2 \frac{1}{(2\nu^2 + 1/2)^2}. \tag{20}$$

The experimental data superimpose to the universal behavior when they are multiplied by a factor of 1.7.

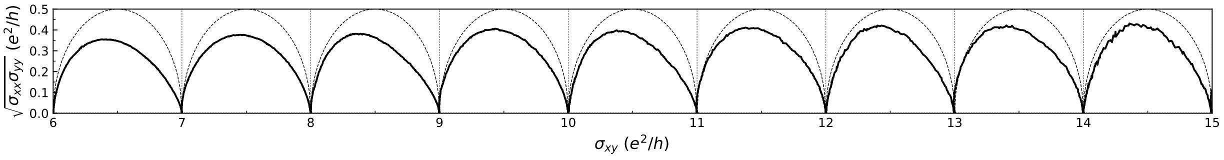

Figure S7. Square root of the product of the longitudinal conductivities, as a function of the transverse conductivity (solid line), as obtained from inverting the resistivity tensor. Notice how the data closely follow the semicircle relation (dashed line): lack of perfect agreement can be attributed to finite-size effects.

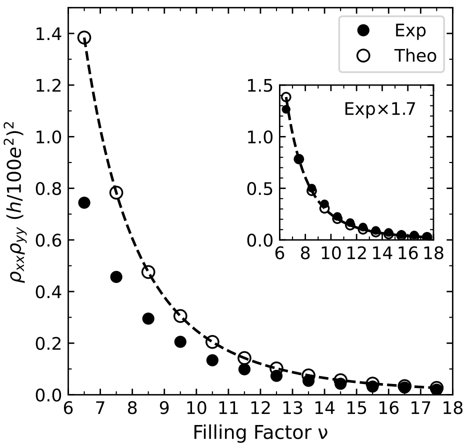

Figure S8. Product of longitudinal resistivities at half-integer filling factors, as obtained from the experimental data of Fig. S5 (solid circles) or from the universal relationship of Eq. 20. Lack of agreement comes from finite-size effects: in the inset it is shown that simply multiplying the experimental data by 1.7 recovers almost exactly the theoretical prediction.

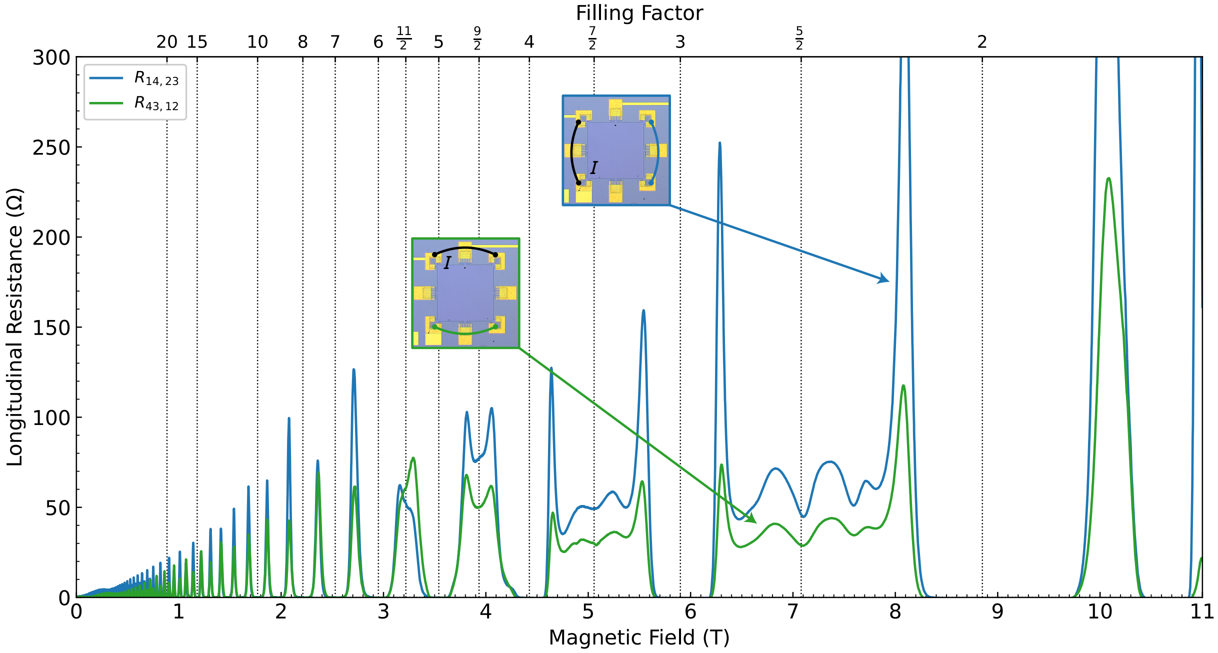

Figure S9. Longitudinal resistance for two pairs of current and voltage probes (following the labeling of Fig. S3), measured on the square patch up to a magnetic field of 11 T.

e. Data at higher magnetic field — For completeness, we report in Fig. S9 the resistance measured with two orthogonal pairs of current and measurement contacts up to 11 T. We notice again the absence of anisotropy at 9/2, in contrast to Refs. [2, 3].

IV. STRIPES ALIGNMENT WITH IN-PLANE MAGNETIC FIELD

The lack of macroscopic stripes alignment which was demonstrated in Sec. III is at variance with the stripes reports in the literature [2, 3]. To experimentally demonstrate that microscopic stripes domain are however present in our material, we align them macroscopically by applying an in-plane magnetic field, which is known to affect the orientation of stripes [15–17].

We measured a 3×3 mm^2 van der Pauw square chip cleaved from the same heterostructure material D170202B (of which the samples reported in the Main text are made), with eight annealed indium contacts at the corners and at the midpoints of the sides of the chip. We applied an in-plane magnetic field by tilting the sample by 30°. Such value was chosen so to have an in-plane magnetic field of about 2 T at filling factor 9/2, a value high enough to macroscopically orient the stripes orthogonal to the in-plane field direction [15–17]. In Fig. S10 (which is the same as Extended Data Fig. 1) we report the longitudinal resistivity measured at 10 mK in the two

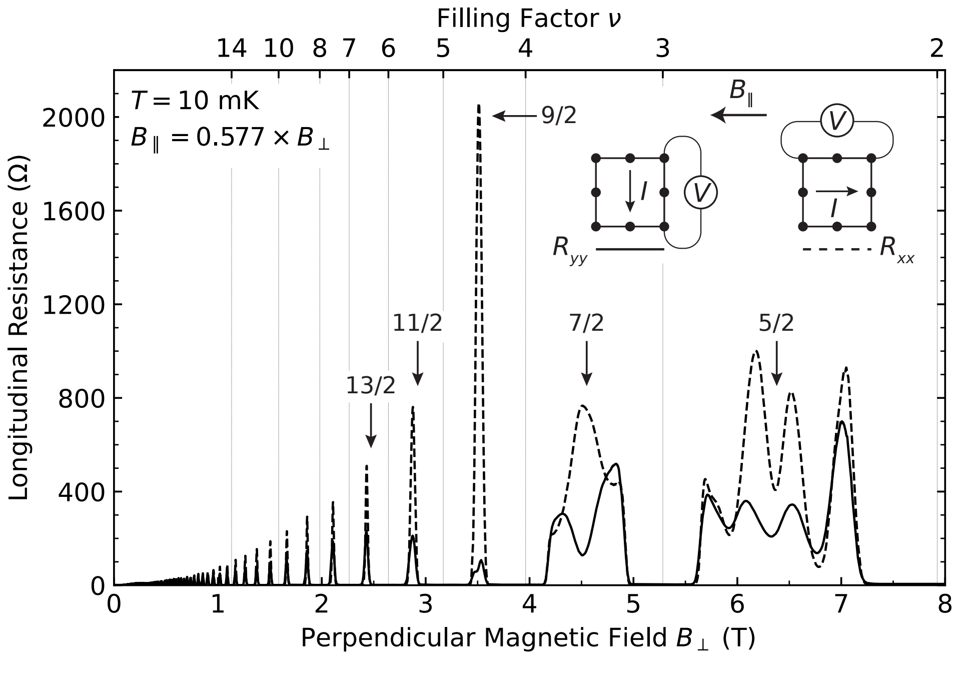

Figure S10. **D170202B** heterostructure — Longitudinal resistance as a function of perpendicular magnetic field $B_\perp$ measured along the two orthogonal directions $\hat{\mathbf{x}}$ (dashed line), parallel to the $(0\bar{1}\bar{1})$ GaAs crystallographic direction, and $\hat{\mathbf{y}}$ (solid line), parallel to $(0\bar{1}1)$. The sample is tilted by 30°, so to apply an in-plane magnetic field $B_\parallel$ in the $\hat{\mathbf{x}}$ direction, which varies linearly with $B_\perp$, and it amounts to about 2 T at filling factor 9/2. The resistance R_{yy} has been multiplied by a factor of 1.5 to match R_{xx} in the low field regime, as conventionally done in the literature. The insets depict the two orthogonal contact schemes.

orthogonal directions, as indicated in the insets. The huge transport anisotropy at 9/2, with large R_{xx} and very small R_{yy}, together with the peak-like shape of R_{xx} and the local minimum of R_{yy}—the accepted transport fingerprints of quantum Hall stripes [2, 3]—provide unmistakable evidence of the macroscopic alignment of stripes at 9/2, and therefore of their microscopic existence in the absence of in-plane field. The transport anisotropy is seen up until $13+1/2$, at about 1.5 T, where it amounts to about a factor of 2, as a consequence of the reduced aligning strength of the in-plane magnetic field, which varies linearly with the perpendicular magnetic field.

To study the melting of the stripe order, we performed measurements at different temperatures of the mixing chamber plate. In Fig. S11 we report the evolution of the longitudinal resistance as a function of magnetic field for the two orthogonal directions, along with the behavior of the R_{xx} maximum and R_{yy} local minimum as a function of inverse temperature. The shape development and the exponential increase (decrease) of the maximum (minimum) as temperature is lowered match again the results reported in the literature [2, 3]. At 200 mK the resistance is isotropic due

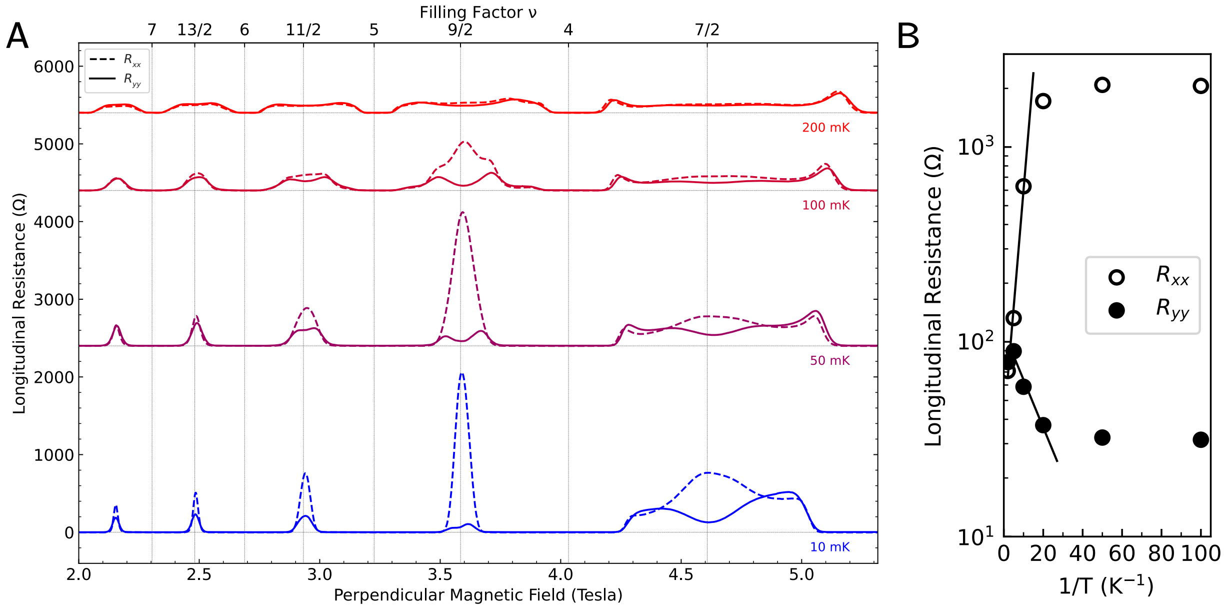

Figure S11. **D170202B** heterostructure — **(A)** Longitudinal resistance as a function of perpendicular magnetic field $B_\perp$ measured along the two orthogonal directions described in Fig. S10 (with the same line styles), applying an in-plane magnetic field $B_\parallel = \tan(30°)B_\perp \simeq 0.577 \times B_\perp$, for different temperatures of the mixing chamber, reported on the right of the plot. The curves referring to different temperatures are vertically offset for clarity. **(B)** Maxima of R_{xx} and local minima of R_{yy} at filling factor 9/2 as a function of inverse temperature in logarithmic scale. We observe a linear increase (decrease) of the maxima (minima) when temperature is lowered.

to the loss of macroscopic alignment. However, we remind that microscopic stripes persist up to about 1 K, as discussed in the Main text.

Additionally, we measured a similar 3×3 mm^2 van der Pauw chip out of the same heterostructure without in-plane magnetic field, and we report the data as a function of temperature in Fig. S12. The behavior is vastly different from the case in which an in-plane field is applied, thus corroborating our claim that stripes do not macroscopically orient without it, as we already demonstrated in Sec. III via a 1×1 mm^2 etched van der Pauw sample.

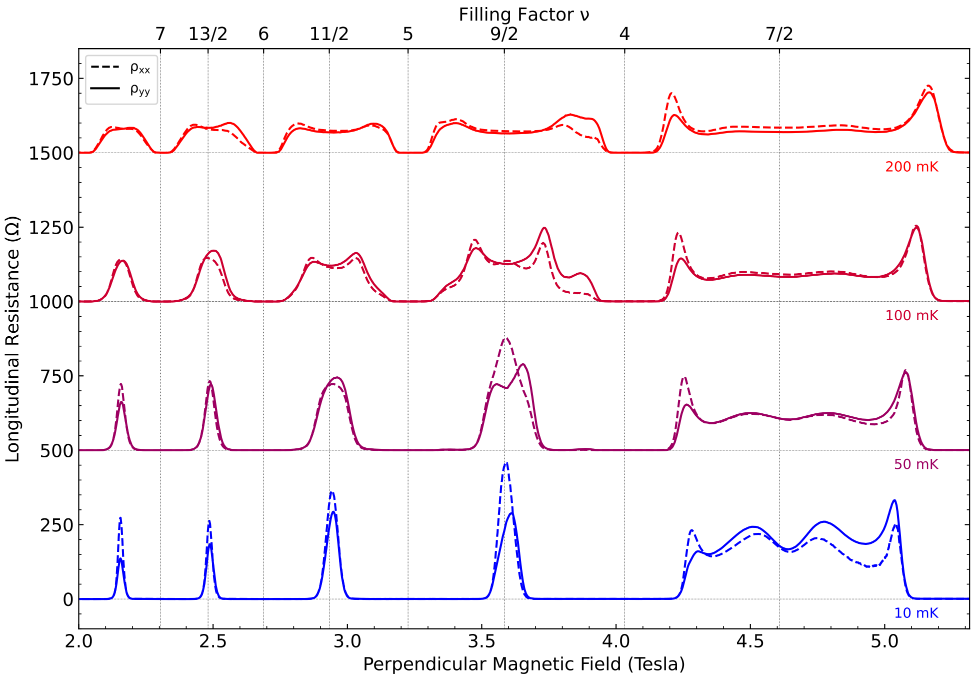

Figure S12. **D170202B** heterostructure — Longitudinal resistance as a function of perpendicular magnetic field $B_\perp$ measured along the two orthogonal directions described in Fig. S10 (with the same line styles), without applying any in-plane magnetic field, for different temperatures of the mixing chamber, reported on the right of the plot. The curves referring to different temperatures are vertically offset for clarity.

V. LONGITUDINAL RESISTIVITY IN THE PRESENCE OF A DENSITY GRADIENT

In Section III we have quantified the inhomogeneous density n_s of the 2DES, and shown that its gradient is related to the length scale of the variation of the Hall resistance, which amounts to about 1.2 mm along the x direction and 0.4 mm along the y direction. Although the Hall bars whose measurements we report in the Main text have $w = 40\,\mu\text{m}$ width and $L = 160\,\mu\text{m}$ length, one order of magnitude smaller than the scale density modulation, which is then usually overlooked in usual quantum Hall measurements, we have applied great care in obtaining the longitudinal resistivity since, being the claimed stripe-ordered phase a charge density modulation, we want to avoid being misled by spurious effects due to other density inhomogeneities. In Fig. S13A,B we report the longitudinal resistance measured at positive magnetic fields on the two sides of both the reference and cavity-embedded Hall bar, $R_{\text{SD},12}, R_{\text{SD},34}$ (where the first two subscripts refer to the injection contacts, and the second two to the voltage probes, according to the labeling of Fig. S13D). It is clear that the two sides do not superimpose, the reason being due to density inhomogeneities, which at magnetic fields above 1 T can impact the current distribution across the sample. As noted in Ref. [4], even a slight density gradient along the Hall bar leads to differences in Hall resistances $R_{\text{SD},24}, R_{\text{SD},13}$, which reflect in differences of $R_{\text{SD},12}, R_{\text{SD},34}$. Indeed, if $V_2 - V_4 > V_1 - V_3$ (a consequence of having a larger n_s in the region between probes 1-3 with respect to 2-4, since the Hall resistance is inversely proportional to n_s, away from plateaux), it immediately follows $V_1 - V_2 < V_3 - V_4$, and also $\Delta R_{\text{long}} = R_{\text{SD},34} - R_{\text{SD},12} = R_{\text{SD},24} - R_{\text{SD},13}$. For example, at 1 T and with $n_s = 4 \times 10^{11}\,\text{cm}^{-2}$ a difference of 1% in density reflects in $\Delta R_{\text{long}} \approx 15\,\Omega$. We have checked that the previous equality holds for both cavity-embedded and reference samples, which also amounts to a sanity check on our measurement setup.

Following Ref. [4], it is straightforward to extract the longitudinal resistivity ρ_{xx}, even when the density gradient is unknown, via the following equation

$$\rho_{xx} = \frac{w}{L} \frac{R_{\text{SD},34} - R_{\text{SD},12}}{\log\left(R_{\text{SD},34}/R_{\text{SD},12}\right)}, \tag{21}$$

a result which follows from the same electrostatic model discussed in Sec. III, and which stays true also when $\rho_{xx} \neq \rho_{yy}$ (the anisotropic case which we are most interested to). We report the extracted resistivity in Fig. S13A-C (in A,B it is still multiplied by the geometric factor $L/w = 4$). Finally, reversing the direction of the magnetic field simply changes the sign of the resistance difference, and it does not affect the value given by Eq. 21.

In complete analogy, we have combined the two measured nonlocal resistances $R_{24,13}, R_{13,24}$ to

obtain the nonlocal resistance we report in the Main text

$$R_{yy} = \frac{R_{24,13} - R_{13,24}}{\log\left(R_{24,13}/R_{13,24}\right)}. \tag{22}$$

Finally, the transverse resistances reported in the Main text are just the average of $R_{\mathrm{SD},24}$, $R_{\mathrm{SD},13}$ or of $R_{24,\mathrm{SD}}$, $R_{13,\mathrm{SD}}$, for ρ_{xy}, ρ_{yx}, respectively.

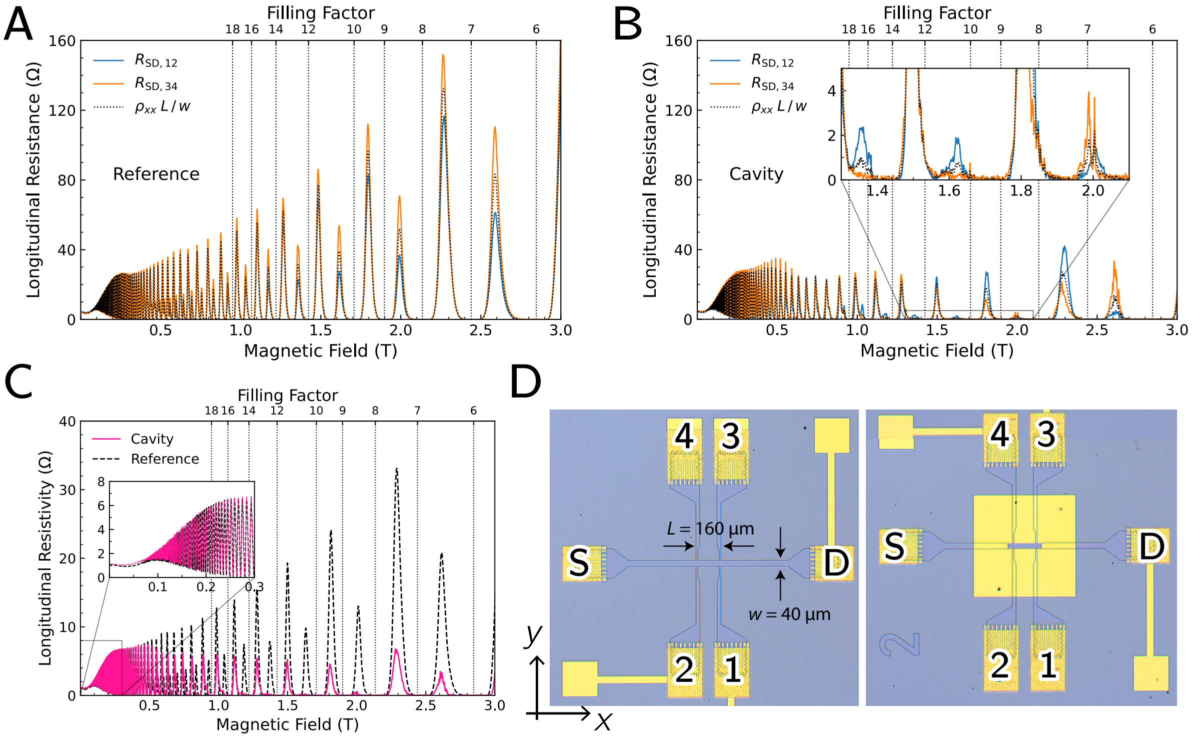

Figure S13. Sample **D170202B-2**—(**A**) Longitudinal resistance measured on the two sides of the reference Hall bar, by passing current through the source-drain contacts (S-D in (D)) and employing voltage probes 1-2 or 3-4 (as labelled in (D)). The dotted line is the longitudinal resistivity (still multiplied by the geometric factor L/w) obtained by combining the measurements on the two sides with the procedure explained in the text. Notice that at all magnetic fields it lies in between them. (**B**) Longitudinal resistance measured on the two sides of the cavity-embedded Hall bar, with the same measurement scheme of (A). In the inset an enlargement of the region where the cavity suppresses the resistivity is displayed. (**C**) Longitudinal resistivity ρ_{xx} compared between cavity-embedded and reference sample. The curves are the same dotted ones of (A, B), divided by the $L/w = 4$. (**D**) Optical microscope pictures of the reference and cavity-embedded Hall bars, with the labeling of the contacts.

VI. NONLOCAL RESISTANCE AND EDGE STATES TRANSPORT MODEL

The longitudinal *resistivity* along the $\hat{\mathbf{x}}$ direction, as presented in Fig 1A in the Main text, is simply obtained via $\rho_{xx} = R_{xx}/4$—given by the ratio between the width w and the length L (i.e. the distance between voltage probes) of the HB (see Fig. 1C in the Main text). In contrast, however, the resistance R_{yy}, especially in small devices like the HB that we use here, cannot be interpreted simply as a *bulk* ρ_{yy}. In particular R_{yy} not only includes contributions from the bulk resistivity in the $\hat{\mathbf{y}}$ direction (i.e. across the stripes in the cavity sample) but also nonlocal contributions from edge states which, in fact, dominate the bulk contributions. Indeed, if one were to simply apply Ohm's law to describe the classical current flow in the HB, one would obtain that the value of R_{yy} would be $\sim 4/\pi\, \rho_{xx} \exp(-\pi L/w) \sim 4 \times 10^{-6} \rho_{xx}$ [18]. The fact that instead we do observe a much larger value of R_{yy} stems from the presence of edge states—a characteristic of the quantum Hall regime—and it is thus termed *nonlocal* resistance [19]. We emphasize that the attribution of R_{yy} to a measure of nonlocal resistance follows directly from the geometry of our HB and is, crucially, independent of the cavity.

In this section we discuss the model to characterize transport at half-integer filling factors, which allows us to obtain from the longitudinal and nonlocal resistances an estimate of the scattering amplitudes into different transport quantum channels. The model is proposed in Ref. [20], and assumes that for filling factors $\nu \in [N-1, N)$, with N a positive integer, transport occurs via $N-1$ dissipationless edge states and a single dissipative bulk channel, decoupled from the other channels, and corresponding to the partially backscattered innermost edge state in the N-th spin-resolved Landau level. We employed the same model in Ref. [21], albeit therein to study the influence of vacuum fields on transport at integer filling factors, in particular by positing that vacuum field-induced backscattering destroys the quantization and gives a finite resistance to the otherwise dissipationless quantum Hall states. Moreover, due to the usage of a more compact Hall bar geometry in the present work, the model can be easily employed to compare the estimated nonlocal resistance with the experimental measurements.

In Fig. S14B we show the schematic employed to define the model: the Hall bar is divided into segments which have geometrical factors $\mathcal{G}$ given by the ratio of their length and width ($\mathcal{G}_1 = 11.5$, $\mathcal{G}_3 = 4$, and $\mathcal{G}_2$ is adjusted to fit the reference data, as detailed below), and which transmit the N-th edge state with probability amplitude t_i, related to the geometrical factor by

$$t_i = \frac{1}{1 + \rho \mathcal{G}_i}, \tag{23}$$

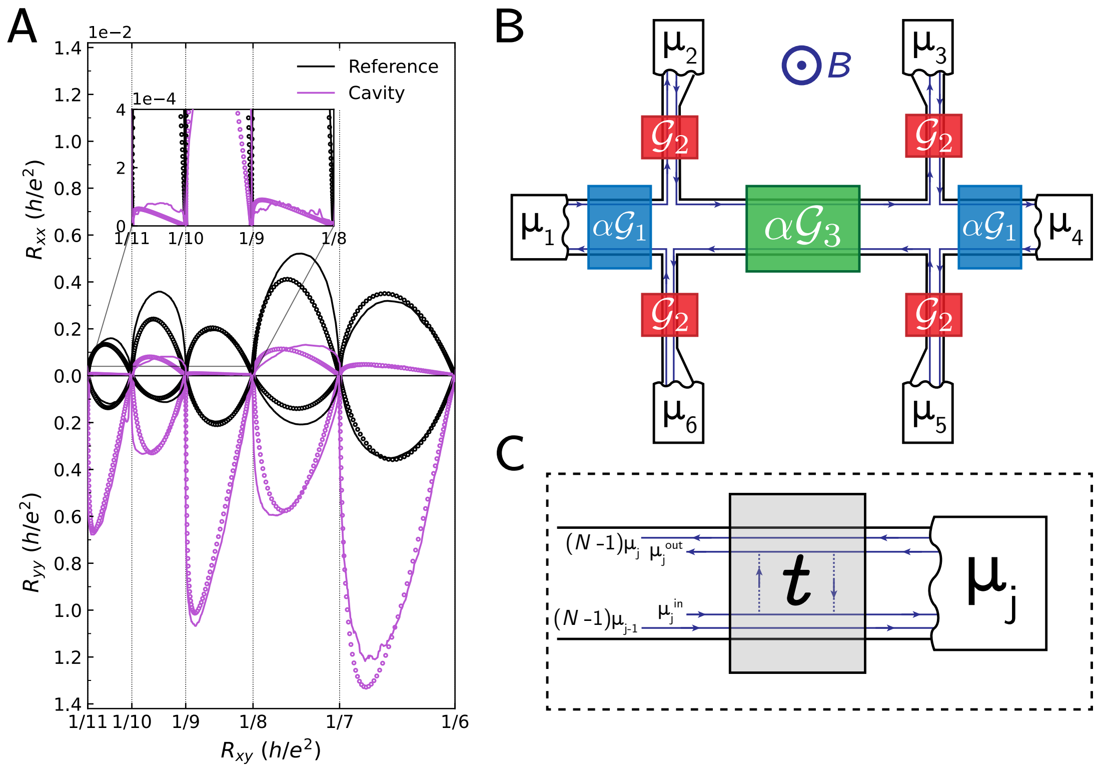

Figure S14. (**A**) Longitudinal (top panel) and nonlocal resistance (bottom panel, with inverted axis) as a function of transverse resistance, for the reference (black solid curve) and cavity-embedded Hall bar (purple solid curve). The black and purple empty circles show the result obtained from the edge state transport model, for the reference and cavity sample, respectively. The inset shows an enlargement between filling factors 8 and 11, where we observe the cavity-induced strong suppression of longitudinal resistance. (**B**) Edge state transport model, showing the geometrical factors $\mathcal{G}_i$ related to the different sections of the Hall bar. We multiply the geometrical factors $\mathcal{G}_1$ and $\mathcal{G}_3$ by a factor $\alpha \ll 1$ to take into account the presence of the stripe-ordered phase, which reduces the probability of scattering from one side to the other. For the isotropic liquid phase $\alpha = 1$. (**C**) Sketch of the scattering process which connects edge states going in opposite directions on the two sides of a section of the Hall bar. While $N-1$ edge states are completely transmitted, the innermost edge state corresponding to the topmost N-th Landau level is partially transmitted, with transmission amplitude t, and partially scattered to the other side, with amplitude $1-t$. The chemical potentials $\mu_j, \mu_{j-1}, \mu_j^{\mathrm{in}}, \mu_j^{(out)}$ which we refer to in the text are indicated.

where ρ is the *resistivity* of the N-th channel only, which is made to vary between zero to infinity as a parameter. In particular, when ρ is zero, the N-th channel behaves as a dissipationless edge channel, and as it increases to infinity the channel is more and more backscattered—with amplitude $1-t$—while the transverse resistance increases from $(1/N)h/e^2$ to $[1/(N-1)]h/e^2$ [22].

Applying Büttiker's multiprobe formula [23] we get the current injected at each contact $j \in [1, 6]$

$$I_j = \frac{e}{h}\left[(N-1)(\mu_j - \mu_{j-1}) - \mu_j^{\text{in}} + \mu_j^{\text{out}}\right], \tag{24}$$

with $\mu_j^{\text{in}}, \mu_j^{\text{out}}$ being the chemical potentials of the innermost channel only (see Fig. S14C), and $\mu_0 \equiv \mu_6$. From current conservation, we have also

$$\mu_j^{\text{out}} = \mu_j t_j + \mu_j^{\text{in}}(1 - t_j), \tag{25}$$

as can be checked in Fig. S14C. Finally, $\mu_j^{\text{in}} = \mu_{j-1}^{\text{out}}$. These $3 \times 6 = 18$ equations in the 18 variables $\mu_j, \mu_j^{\text{in}}, \mu_j^{\text{out}}$ can be solved after providing as boundary conditions the current injected in the leads I_j (for example, to measure the longitudinal resistance we inject current I through contacts 1 and 4, so $I_1 = -I_4 = I$), and we have to fix one value of the chemical potentials as a reference (e.g. $\mu_4 = 0$). After solving the linear system, we can directly obtain the longitudinal, nonlocal, and transverse resistances, and we compare them to the experimental data in Fig. S14A (the experimental data have been combined to remove effects of density inhomogeneities as detailed in Section V). To model the transport in the stripe-ordered phase, we multiply the geometrical factors $\mathcal{G}_1, \mathcal{G}_3$ by a factor $\alpha < 1$, intended to represent the preferential direction of transport along rather than orthogonal to the stripes ($\alpha = 1$ in the isotropic liquid phase of the reference sample). In Table S2 we report the parameters employed to fit the model to the experimental data measured on the cavity sample. The geometrical factor $\mathcal{G}_2$ was fitted first to the experimental data of the reference sample and then not changed in the fitting of the cavity one. In the table we report also the obtained values of the transmission amplitudes, as given by Eq. 23, for the reference sample in the isotropic liquid phase (t_l obtained using $\mathcal{G}_3$, with $\alpha = 1$), and the cavity sample in the stripe-ordered phase (t_s obtained using $\mathcal{G}_3$, multiplied by α detailed in the table). Considering the backscattering amplitudes, we obtain values of $1 - t_s$ one order of magnitude smaller than $1 - t_l$ at filling factors $2N + 1/2$.

ν	$\mathcal{G}_2$	α	t_l	t_s
6+1/2	18	0.04	0.75	0.94
7+1/2	6	0.07	0.75	0.88
8+1/2	18	0.01	0.75	0.97
9+1/2	7	0.10	0.75	0.87
10+1/2	18	0.01	0.75	0.97

Table S2. Parameters employed to fit the model to the experimental data for the cavity sample, and values obtained for the transmission amplitude in the isotropic liquid and stripe-ordered phases (t_l, t_s, respectively).

VII. EXPERIMENTAL REPRODUCIBILITY

A. Reproducibility across different magnetic field sweeps

As stated in the Main text, we have measured both reference and cavity-embedded Hall bars in the same cool-down, and simultaneously during several magnetic field sweeps, employing multiple synchronized lock-in amplifiers. In particular, we show in Fig. S15 the measurements for 4 successive sweeps at positive and negative magnetic field, and sweeping the magnetic field from zero to its maximum value or vice-versa. We display the results by showing the longitudinal resistivity ρ_{xx} and nonlocal resistance R_{yy} as a function of transverse resistivity ρ_{xy}, to better highlight the variations away from the quantized plateaux. We notice that across different sweeps the curves compare fairly well. A plethora of details can still be noticed, such as the slanted shape of the peaks, which seems to partially depend on the sign of the field and direction of the sweep, but this analysis goes far beyond the purpose of the present work.

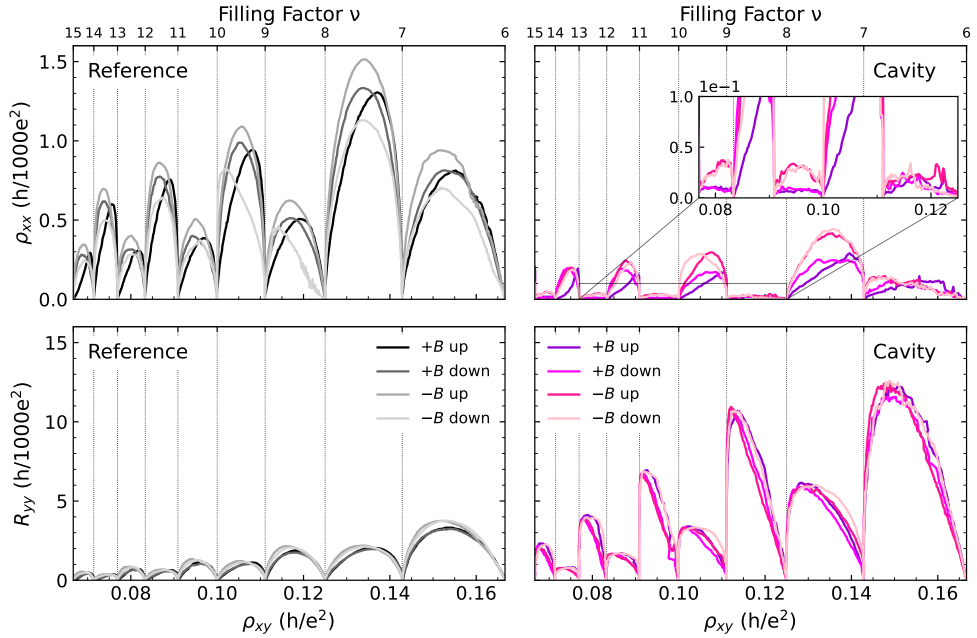

Figure S15. Sample **D170202B-2**—Longitudinal resistivity ρ_{xx} (top panels) and nonlocal resistance R_{yy} (bottom panels, notice the different y-axis scale) as a function of the transverse resistivity ρ_{xy} for the reference (left panels) and cavity-embedded (right panels) Hall bars, for different magnetic field sweeps, that is going from 0 to 3 T ($+B$ up), from 3 to 0 T ($+B$ down), from 0 to -3 T ($-B$ up), and from -3 to 0 T ($-B$ down), displayed with colors according to the legends.

B. Measurement on a rotated cavity-embedded Hall bar

In Figure S16 we compare the longitudinal resistivity measured on two cavity-embedded Hall bars fabricated on the same chip (Sample **D170202B-1**, see Section VII D 1) but rotated by 90° with respect to each other. This is done as a further confirmation that the effect follows the polarization of the cavity vacuum field. Indeed, we observe a similar resistivity suppression in the two cavity samples, as compared to the reference one. The difference in the magnitude of the resistivity suppression comes from the different profile of the cavity edges, as discussed in Section VII C.

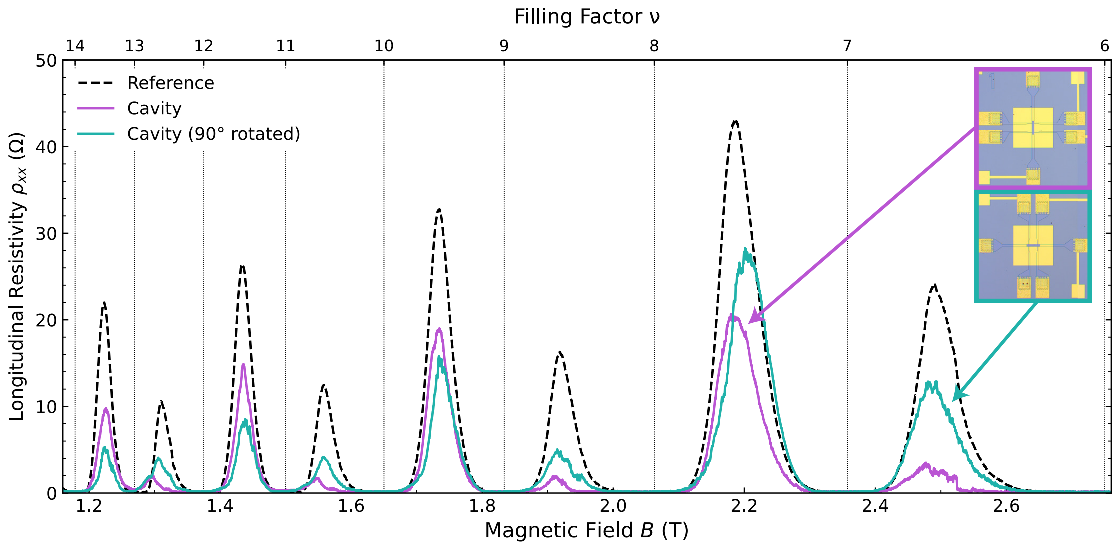

Figure S16. Sample **D170202B-1**—Longitudinal resistivity as a function of magnetic field for a reference HB (black dashed line), and for two cavity-embedded HBs fabricated on the same chip but rotated by 90° with respect to each other (colored solid lines). The inset shows an optical microscope picture of the chip, showing the orientation of the two cavity samples. We observe a similar longitudinal resistivity suppression within the cavity samples—with respect to the reference one—which supports the fact that it is the cavity field orientation to define the collective alignment axis of the stripe order.

C. Role of the cavity edges

In Figure S17 we explain the effect of the cavity edges on the magnitude of the measured magnetotransport anisotropy. We emphasize that the in-plane distance between the 2DES and the cavity edge is about 250 nm, as marked directly on the figure. In particular, we considered two cavity designs: the slot antenna already discussed in the Main text, and the complementary split-ring resonator (CSRR) which was employed in Refs. [1, 21]. The two designs are fabricated on

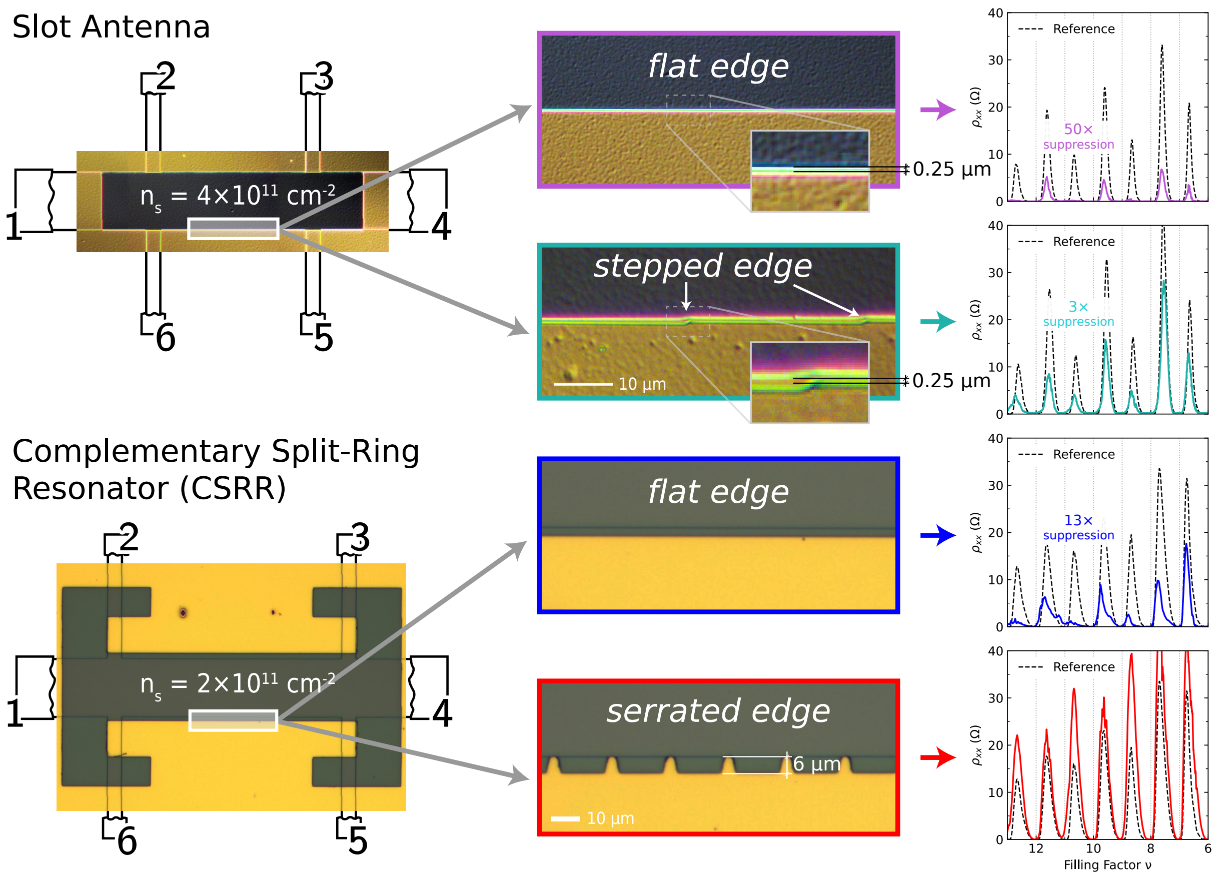

Figure S17. Impact of the cavity edges flatness on the magnitude of the longitudinal resistivity suppression in a cavity-embedded Hall bar. Two different designs of the cavity were employed: the slot antenna which is the object of the present work (top), and the complementary split-ring resonator which was employed in Refs. [1, 21] (bottom). For each cavity, two variations have been fabricated, one with flat edges and one with stepped or serrated edges. The four panels on the right report the longitudinal resistivity as a function of filling factor for the respective cavity (colored solid lines) and reference samples (black dashed lines). Notice that the 2DESs have different densities, as indicated on the optical microscope pictures.

different chips, where the 2DES have densities $4 \times 10^{11}\,\mathrm{cm}^{-2}$, and $2 \times 10^{11}\,\mathrm{cm}^{-2}$, respectively, and have been realized in two variations, one with flat cavity edges and one with stepped or serrated edges. On each chip a reference sample is also present. By comparing the different longitudinal resistivities, we observe that the flat edges provide the largest resistivity suppression in the cavity-embedded samples, with a larger suppression in the sample having higher 2DES density. When employing instead a cavity with serrated edges, we clearly see that the resistivity suppression is absent. We did not attempt to quantify the effect further or to relate it to the particular spatial profile of the electromagnetic field mode, which could constitute a topic for further research. Here and in the following, we attribute the better resistivity suppression in the slot antenna as compared

to the CSRR to the geometry of the slot antenna that forces modes with the field along the current to higher frequencies.

D. Reproducibility across different samples

In this section we report measurements on other samples processed also on different heterostructures, where we observed a magnetotransport anisotropy comparable to the one reported in the Main text. The indication inside the parentheses is the internal labeling of the fabricated samples.

1. Second sample processed on D170202B (D170202B-1)

We fabricated the sample presented in the Main text and this one on the same chip, and then cleaved it afterwards. The only difference thus comes from the slightly lower density gradient, due to the different physical position on the heterostructure wafer (the photolitography mask is identical). In Fig. S18 we report the comparison between longitudinal resistivities of the reference

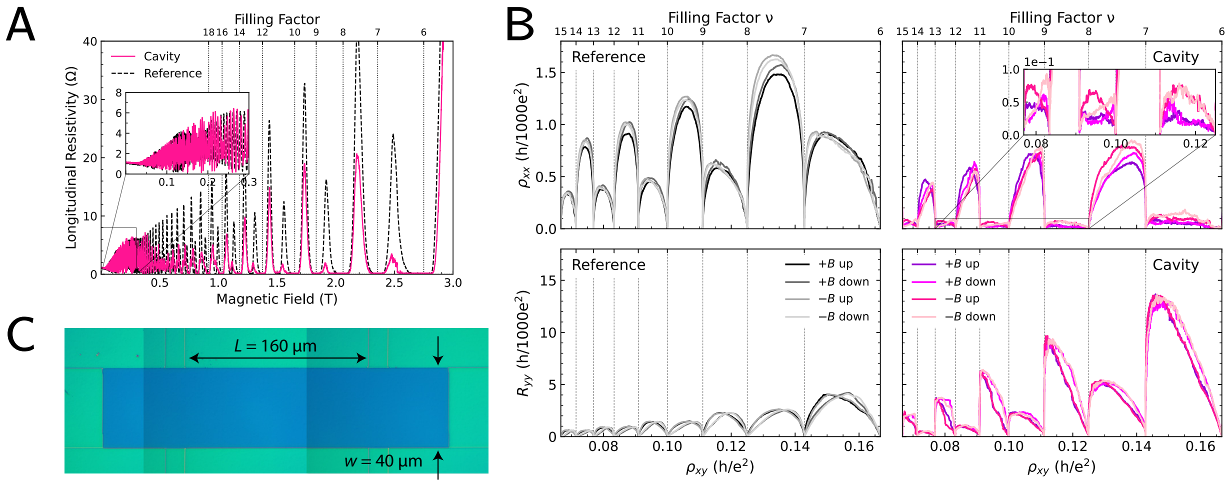

Figure S18. Sample **D170202B-1**—(**A**) Longitudinal resistivity measured for a reference and a cavity-embedded Hall bar, as a function of magnetic field. Around filling factor $8 + 1/2$ we observe a ratio of about 9.5. (**B**) Longitudinal resistivity ρ_{xx} (top panels) and nonlocal resistivity R_{yy} (bottom panels, notice the different y-axis scale) as a function of the transverse resistivity ρ_{xy} for the reference (left panels) and cavity-embedded (right panels) Hall bars, for different magnetic field sweeps, that is going from 0 to 3 T ($+B$ up), from 3 to 0 T ($+B$ down), from 0 to -3 T ($-B$ up), and from -3 to 0 T ($-B$ down), displayed with colors according to the legends. Notice how across different sweeps the results are fairly comparable. (**C**) Optical microscope picture of the slot antenna resonator.

and cavity-embedded Hall bars, showing clearly the cavity-suppressed resistivity at half-integer filling factors $2N + 1/2$, with N ranging from 3 to 7. We also report the reproducibility study over different magnetic field sweeps (changing sign and ramp direction), assessing again the robustness of the cavity-induced magnetotransport anisotropy.

2. Sample processed on D170209B (D170209B-1-40B)

This sample was one of the very first where the suppression of resistivity at half-integer filling factors in the cavity-embedded Hall bar was observed. It was processed and first measured in 2018, and then remeasured in 2023, with a vastly updated experimental setup: we changed the superconducting Helmholtz coils magnet, capable of reaching 6 T, with a solenoidal superconducting magnet, capable of reaching 12 T; we remade all electrical wiring and changed chip socket; we changed the capacitors of the 100 kHz low-pass filter from 10 to 1 nF, and increased accordingly the resistors from 1 kΩ to 10 kΩ. Moreover, the 2018 measurement is performed with asymmetrically injected 3 nA current, while in 2023 10 nA where injected symmetrically by employing two 100 MΩ resistors before both source and drain. The heterostructure contains a 27 nm-wide square quantum well, with a 60 nm spacer from the doping layer, and the 2DES has density $4.2 \times 10^{11}\,\mathrm{cm}^{-2}$ and mobility $2.5 \times 10^{7}\,\mathrm{cm}^{2}\mathrm{V}^{-1}\mathrm{s}^{-1}$ (measured at 1.3 K without illumination). The Hall bar has still a width of 40 µm, and the voltage probes are 160 µm apart, however, the overall length is about 1.4 mm (since it was probed at different positions), making it harder to model and understand nonlocal measurements. In Fig. S19 we report the longitudinal resistivity as a function of magnetic

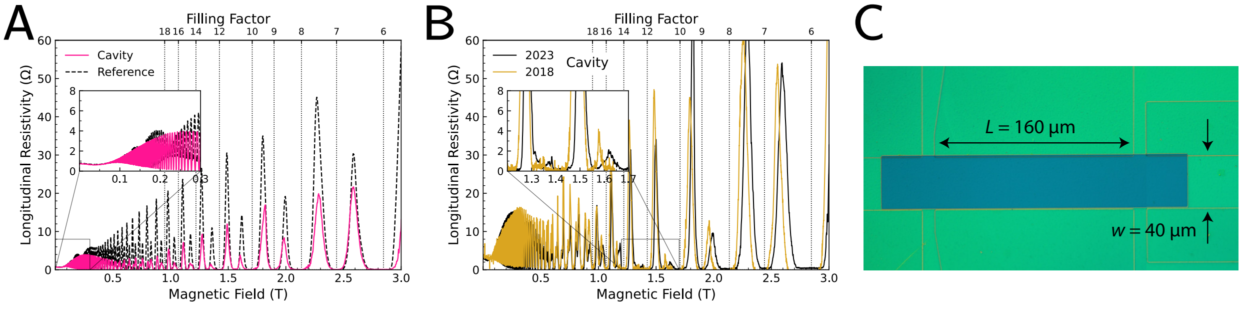

Figure S19. Sample **D170209B-1-40B**—(**A**) Longitudinal resistivity measured for a reference and a cavity-embedded Hall bar, as a function of magnetic field. Around filling factor $12 + 1/2$ we observe a ratio of about 8. (**B**) Comparison between the longitudinal resistivity (obtained by taking into account only one side of the Hall bar) of the cavity-embedded sample as measured in 2018 and in 2023, with a vastly updated experimental setup. (**C**) Optical microscope picture of the slot antenna resonator.

field, where we can clearly distinguish the resistivity suppression in the cavity sample for half-integer filling factors between $10 + 1/2$ and $14 + 1/2$. We also display a plot comparing the longitudinal resistivity as measured with the old setup in 2018, and with the new one in 2023, showing perfect agreement.

3. Sample processed on D200923A (D200923-Scs#1)

This sample was processed on a different heterostructure and employing a different design of the resonator with respect to the one presented in the Main text. The heterostructure contains a 27 nm-wide GaAs/$Al_{0.14}Ga_{0.86}As$ square quantum well, with a 80 nm spacer from the doping layers, and it is overall located 280 nm below the surface. The 2DES has density 3.2×10^{11} cm^{-2} and mobility 2.2×10^7 cm^{2}V^{-1}s^{-1}, when measured at 1.3 K without illumination, and 1.9×10^7 cm^{2}V^{-1}s^{-1} as assessed from the Hall bar measurement at millikelvin temperatures. The cavity is a complementary split-ring resonator (CSRR) [24], and the Main difference with the slot antenna resonator comes from the longer length of the current path around the capacitor gap (provided by the arms' shape), which makes the inductance larger. We have fabricated two CSRRs, with resonance frequencies 115 GHz and 135 GHz, and normalized coupling 45% and 39%, respectively. Since the resonant frequency is related to the length of the resonator (288 µm and 230 µm, respectively, as indicated in Fig. S20B), we have scaled the distance between the probes L accordingly, and fabricated also two

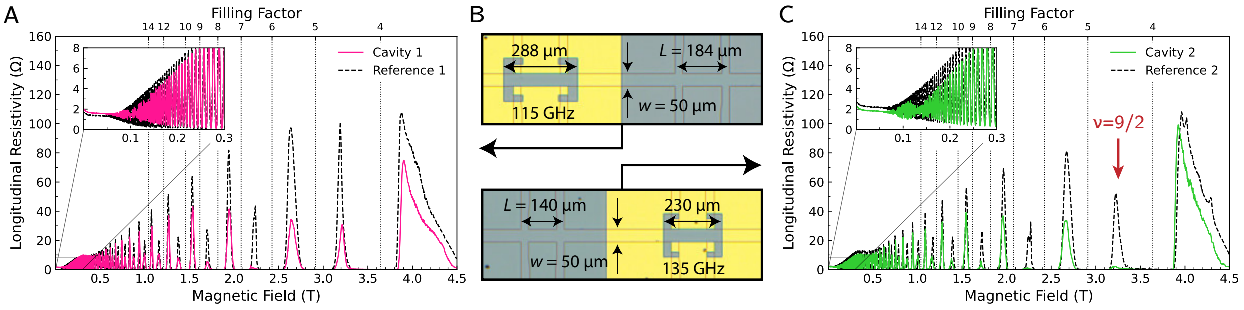

Figure S20. Sample **D200923-Scs#1**—(**A**) Longitudinal resistivity measured for a reference and a 115 GHz cavity-embedded Hall bar, as a function of magnetic field. Around filling factor $6 + 1/2 = 13/2$ we observe a ratio of about 21. (**B**) Optical microscope pictures of the two pairs of reference and cavity-embedded resonators. The first pair (top, data are presented in (A)) has distance between voltage probes of 184 µm, while the second one (bottom, data are presented in (C)) has distance between voltage probes of 140 µm. Both Hall bar pairs have width 50 µm. (**C**) Longitudinal resistivity measured for a reference and a 135 GHz cavity-embedded Hall bar, as a function of magnetic field. For filling factors $6+1/2 = 13/2$ and $4+1/2 = 9/2$ we observe a more than 20-fold cavity suppression of the resistivity.

different reference Hall bars, with the same voltage probe distance as the respective resonator. The Hall bar width is 50 µm (see Fig. S20B for the microscope pictures with annotated dimensions). We report in Fig. S20A,C (Fig. S20 is the same as Extended Data Fig. 3) the longitudinal resistivities for both pairs of reference and cavity-embedded Hall bars, and one can notice the cavity-induced suppression of the resistivity peaks at filling factor $6 + 1/2 = 13/2$ being more than a factor of 20.

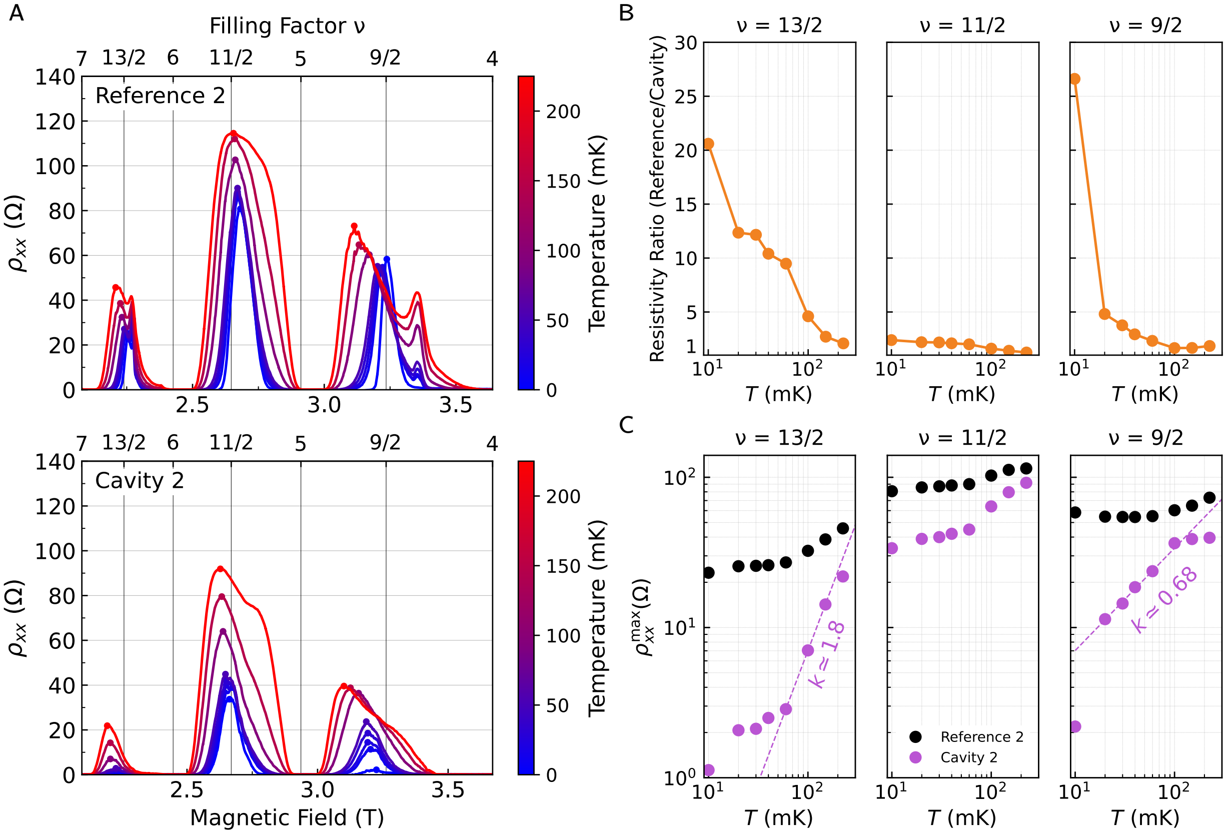

Figure S21. Sample **D200923-Scs#1**—(**A**) Longitudinal resistivity ρ_{xx} as a function of magnetic field for the reference (top) and 135 GHz cavity-embedded HB (bottom), measured at different temperatures of the mixing-chamber plate (colors according to the color bar on the right). At the lowest temperature the data are the same as presented in Fig. S20C. (**B**) Ratio between the resistivity maxima measured in the reference and in the cavity-embedded HBs as a function of temperature, for filling factors $13/2, 11/2, 9/2$. The data are taken from the plots in panel **A**, where the maxima at different temperatures are indicated with circle markers. (**C**) Longitudinal resistivity maxima at half-integer filling factors as a function of temperature, in log-log scale. Again, the data are taken from the plots in panel **A**. Black and purple colors refer to the reference and cavity sample, respectively. The resistivity maxima at $\nu = 13/2, 9/2$ of the cavity sample follow a power-law behavior as a function of temperature, $\rho_{xx}^{\max} \propto T^k$, with k indicated in the subplots.

For both cavity-embedded Hall bars we observe a similar suppression of the resistivity peak also at filling factor $4 + 1/2 = 9/2$, where the quantum Hall stripe phase is the most robust [2, 3, 25]

(the suppression is much stronger in the 135 GHz cavity, which is smaller than the 115 GHz one). As explained in the Main text, the cavity-induced suppression happens in a range of magnetic fields for which the cyclotron frequency ω_c is in a frequency range in which the cavity modes show anisotropy. Since this sample has a lower carrier density this magnetic field range thus corresponds to lower filling factors, including 9/2.

As it is done in the Main text for the sample processed on the D170202B heterostructure, we perform a temperature study of the longitudinal resistivity of the 135 GHz cavity-embedded sample, and we report the results at filling factors between 4 and 7 in Fig. S21. As already discussed in the Main text, the cavity-induced alignment is mainly present at filling factors $2N + 1/2$, i.e. at $4 + 1/2 = 9/2$ and $6 + 1/2 = 13/2$, and the resistivity ratio follows a similar decay with increasing temperature as it is shown in Fig. 2C in the Main text. Moreover, we notice a similar power-law increase of the resistivity maxima in the cavity-embedded sample as already reported in Fig. 2D in the Main text, albeit with a different exponent. As already discussed above, we are focusing here on the cavity-induced alignment in a similar range of magnetic fields as the one presented in the Main text, but which correspond to lower filling factors since the density of this sample is lower than the one in the Main text.

4. Sample processed on D151202B (D151202B)

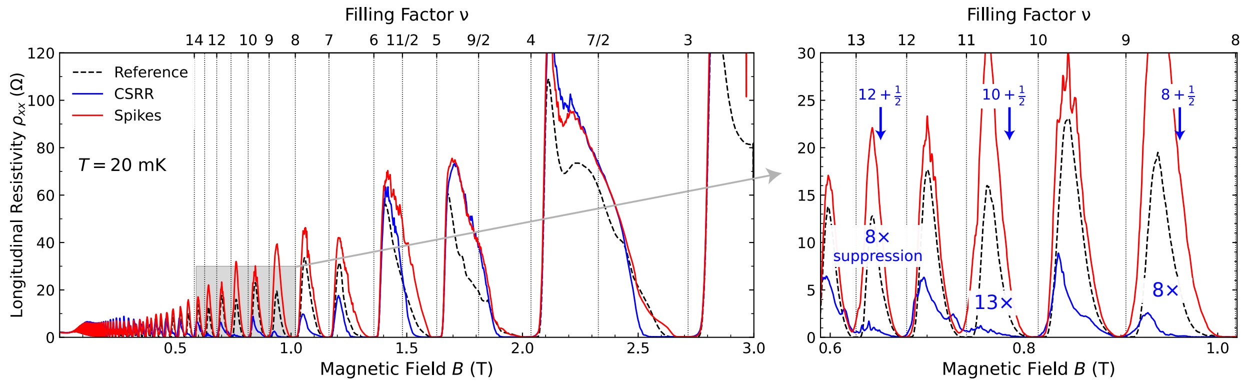

Figure S22. Sample **D151202B**—Longitudinal resistivity as a function of magnetic field, for the reference HB (black dashed line), the complementary split-ring resonator cavity-embedded HB ("CSRR", blue solid line), and the variation of the latter having serrated edges ("Spikes", red solid line)—see the microscope picture of Fig. S17, bottom panel. The right panel is an enlargement of the grey rectangle highlighted on the left panel. We observe that while the resistivity in the flat edges cavity-embedded sample is about an order of magnitude lower than the reference one, in the serrated edge cavity the effect is absent.

Also this sample was processed on another different heterostructure, which contains a 30 nm-wide square quantum well, with a 100 nm spacer from the doping layer, and the 2DES has density $2.1 \times 10^{11}\,\mathrm{cm}^{-2}$ and mobility $1.7 \times 10^{7}\,\mathrm{cm}^2\mathrm{V}^{-1}\mathrm{s}^{-1}$ (measured at 1.3 K without illumination). The cavity design is again a CSRR, with 180 GHz resonance frequency of the fundamental mode. On this sample we also fabricated a cavity having serrated edges, to investigate the role of edge flatness, as discussed in Section VII C. As already discussed therein, we observe a similar resistivity suppression in the HB embedded in the cavity having flat edges, which is in contrast absent in the sample within the cavity having serrated edges.

5. Sample processed on D151202B, investigated in Ref. [1]

The heterostructure from which this sample was fabricated is the same one of Section VII D 4. The details on the cavity design and measurement technique are reported in detail in Ref. [1]. Here we limit ourselves to report in Figure S23 the longitudinal resistivity as a function of magnetic field at values relevant for the observation of the stripe-ordered phase. We indeed see—to a small extent—that reducing the distance between the hovering cavity and the 2DES, i.e. increasing the light-matter coupling, the resistivity peaks at half-integer filling factors reduce in amplitude. We attribute the way smaller effect to the fact that even at the closest position the cavity is still about 0.6 µm away from the 2DES (summing the 0.35 µm distance of the cavity from the sample surface and the 0.23 µm depth of the 2DES), while by directly evaporating the cavity on top of the sample, as done in the present work, we can almost completely embed the 2DES inside it, thus increasing the coupling to the anisotropic vacuum fluctuations. Moreover, the alignment precision of the hovering cavity edges is about 5 µm, much too coarse with respect to the precision of the evaporated one (within 0.3 µm, see Sec. VII C), which leads to the dramatic effect reported in the Main text.

Another difference worth mentioning is the 2DES density, which is $4 \times 10^{11}\,\mathrm{cm}^{-2}$ in the present work, and $2 \times 10^{11}\,\mathrm{cm}^{-2}$ in Ref. [1]. While the lower density should render the microscopic stripe domains more stable (due to the higher magnitude of the exchange energy), the effect on the macroscopic alignment stability may be different, but we did not investigate it.

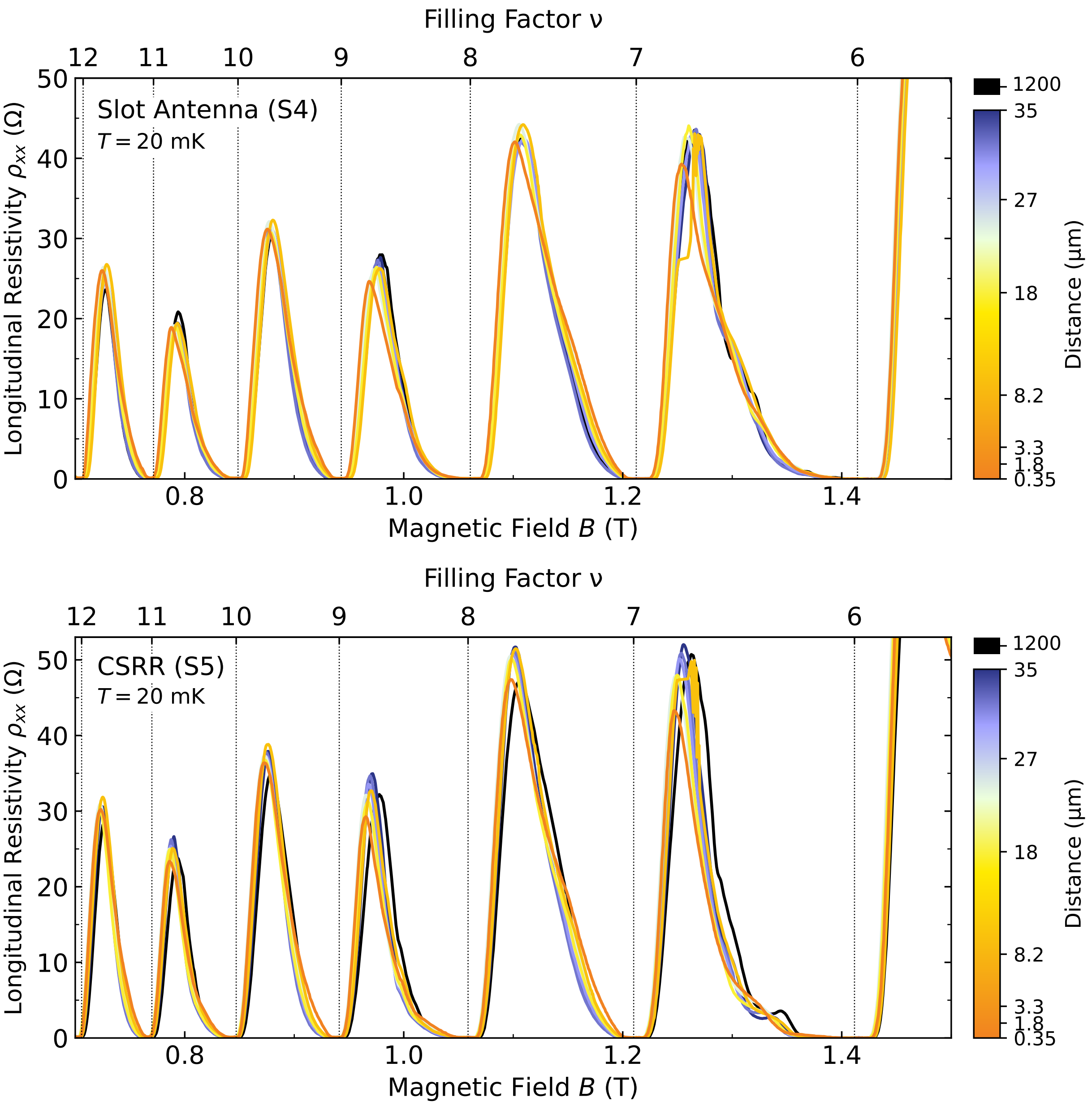

Figure S23. Sample of **Ref. [1]**—Longitudinal resistivity as a function of magnetic field measured in the hovering cavity experiment reported in Ref. [1]. Curves with different colors refer to the different distances between the hovering cavity and the 2DES, according to the color bar on the right. The top panel refers to the slot antenna cavity, while the bottom one to the complementary split-ring resonator.

VIII. TEMPERATURE STUDY OF THE ODD-INTEGER FILLING FACTOR MINIMA

In this section we discuss the thermally activated behavior of resistivity minima at odd-integer filling factor, which allows us to estimate the Zeeman energy gap, and to show that in the cavity sample this is strongly reduced with respect to the reference one. This confirms our previous results reported in Ref. [1], and we also comment on the connection with the results of Ref. [21]. The wealth of effects which we observe in cavity-embedded samples at different filling factor regimes requires indeed that our results are consistent with each other.

In Ref. [21] we experimentally demonstrated in a Hall bar embedded in a similar subwavelength cavity how the nonlocal nature of vacuum fields could provide, via a vacuum-induced scattering mechanism [26], a finite resistivity to the otherwise zero-resistance states at high integer filling factors, affecting in particular the odd integer ones. The latter refer indeed to the case in which the Fermi energy lies within Zeeman split states, whose energy gap ΔE_{Zeeman} is much smaller than the cyclotron gap, separating instead occupied and empty states at even integer filling factor regimes.

In Ref. [1] we further understood that the vacuum-induced scattering—within a single-particle description—induces an effective cavity-mediated electron-electron interaction—at the many-body level— which affects the magnitude of ΔE_{Zeeman} and strongly reduces it in the odd integer filling factor regime (a behavior already observed in Ref. [21]). We can express the energy gap as a function of magnetic field B as

$$\Delta E_{\text{Zeeman}} = g\mu_B B - \mathit{\Gamma}, \tag{26}$$

where g is the effective electronic g-factor, which is renormalized by electron-electron interactions [27], μ_B is Bohr's magneton, and $\mathit{\Gamma}$ is a parameter that takes into account the Landau level broadening due to disorder [28]. Studying the temperature dependence of the longitudinal resistivity minima ρ_{xx}^{ν} at the odd integer filling factors we can obtain an estimate of ΔE_{Zeeman}: indeed, ρ_{xx}^{ν} are thermally activated following

$$\rho_{xx}^{\nu}(T) = \rho_{xx}^{\nu}(0)e^{-T_{\text{act}}/2T} = \rho_{xx}^{\nu}(0)e^{-E_{\text{act}}/2k_B T}, \tag{27}$$

where k_B is Boltzmann's constant, T is the temperature, and the activation energy, equivalent to the mobility gap, $E_{\text{act}} = k_B T_{\text{act}} = \Delta E_{\text{Zeeman}}$. The reason for the factor of 1/2 comes from the law of mass-action, prescribing that the number of electrons excited to the empty Zeeman split state above the Fermi energy is equal to the number of holes left behind in the Zeeman split state below it.

In Fig. S24 we report the longitudinal resistivity minima at odd-integer filling factors between 11 and 21 as a function of inverse temperature, for both reference and cavity samples. The minima follow a temperature activated behavior following Eq. 27 for temperatures above 0.1 K, and by a fitting procedure we obtain the activation energy (temperature) which we report in Fig. S25, as a function of magnetic field. We observe how the activation energy for the cavity-embedded sample is consistently lower than the one of the reference sample. Moreover, while at low temperatures the resistivity minima for the cavity sample are higher than the one of the reference (as it was demonstrated in Ref. [21]), increasing the temperature we observe the opposite. By fitting the activation energy to Eq. 26, we can extract the value of the g-factor, which is strongly suppressed in the cavity with respect to the value in the reference. This result is consistent with what was reported in Ref. [1], the absolute values being lower due to the higher electron density of the 2DES [29].

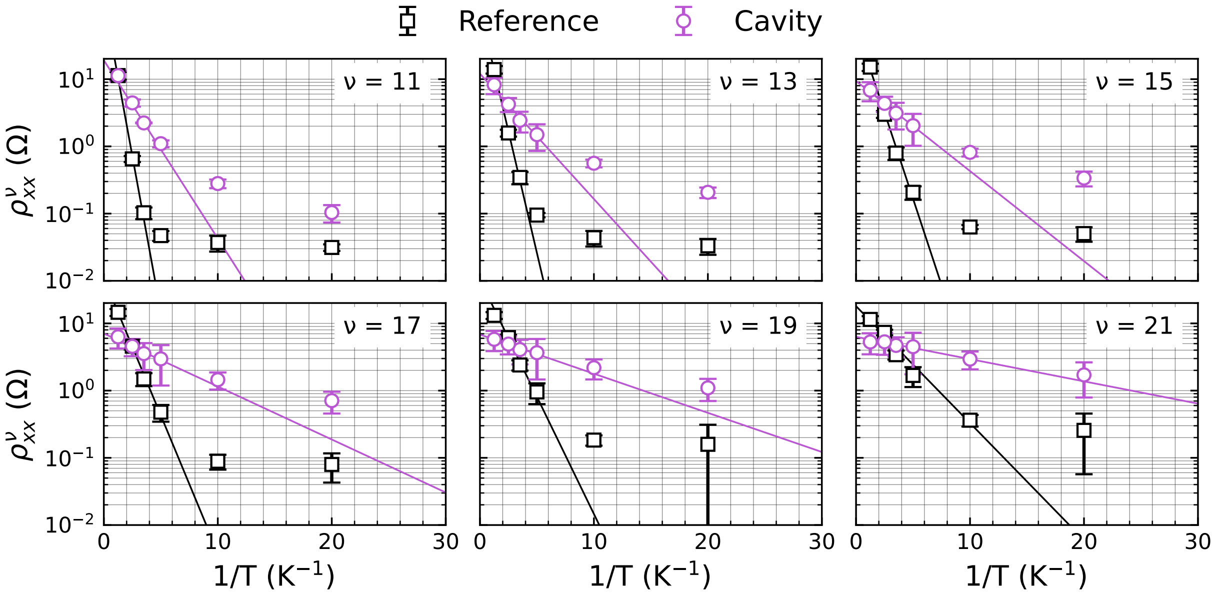

Figure S24. Longitudinal resistivity minima at odd-integer filling factors between 11 and 21 as a function of inverse temperature, for both the reference (empty square markers) and cavity (empty round markers) samples. For temperatures above 0.1 K the resistivity minima display an activated behavior following Eq. 27, which is fitted via the solid black and purple lines, for the reference and cavity sample respectively. The data are obtained as an average between both positive and negative magnetic field (after putting together measurements obtained on the two sides of the Hall bar, as explained in Sec. V) and both up and down field sweep directions, and the error bars represent their standard deviation.

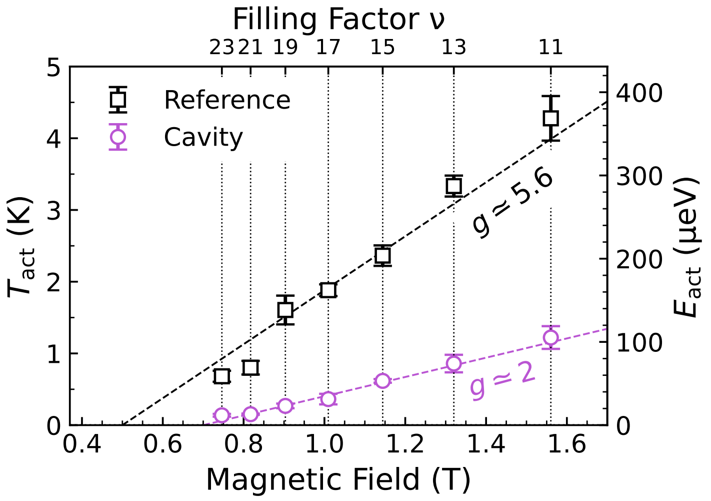

Figure S25. Activation temperature (energy on the right axis) as a function of magnetic field at odd integer filling factors for both reference (black empty squares) and cavity sample (purple empty circles). Notice how the slope, which is proportional to the g-factor, is vastly suppressed in the cavity, linking the present work to the findings of Ref. [1]. The error bars represent the standard deviation of the Arrhenius law fit.

A point deserving further investigation is the relationship between the suppression of the g-factor and the different behavior of the reference-to-cavity resistivity ratio observed at half-integer filling factors $\nu = 2N + 1/2$ and $\nu = (2N + 1) + 1/2$, which we connected in the Main text to the different occupation of the spin-split Landau levels (see Fig. 1E in the Main text). Specifically, in samples having lower electron density we have observed a lower extent of the g-factor suppression at odd integer filling factors, connected with a less pronounced suppression of the resistivity at half-integer filling factors, which could indicate a relationship between the two, not currently taken into account in the present theoretical interpretation.

As a technical note, the temperature was changed by passing current in a $120\,\Omega$ resistor attached to the mK plate of the dilution refrigerator, and controlling the measured temperature via a PID feedback loop, allowing us to change it between 20 and 800 mK. For temperatures below 50 mK the reading at finite magnetic field is not reliable, due to the magnetoresistance of the temperature sensor.

IX. THEORETICAL INTERPRETATION

Our goal in this section will be to calculate the free energy of the system for different orientations of quantum Hall stripes in the presence of the vacuum fluctuations of the slot antenna cavity. This will permit us to compute the anisotropy *per electron* induced by the interplay of electromagnetic vacuum fluctuations and the quantum Hall stripes, helping us determine how the stripes should align.

A. Generic Formulation

We begin by establishing a general formalism for calculating the perturbative contribution to the free energy arising from the coupling of electronic degrees of freedom and electrodynamic fluctuations of the slot antenna cavity, working within the imaginary time Matsubara formalism [30]. We consider a generic two-dimensional electron system (2DES) characterized by a current density $\mathbf{J}(\mathbf{r}, \tau)$, coupled to the THz cavity induced electromagnetic field described by the vector potential $\mathbf{A}(\mathbf{r}, t)$. The interaction between this current and the electromagnetic field can be modeled using the Matsubara action:

$$\mathcal{S}_{\text{int}} = \int_0^\beta d\tau \int d^2\mathbf{r}\, \mathbf{J}(\mathbf{r}, \tau) \cdot \mathbf{A}(\mathbf{r}, \tau), \tag{28}$$

where $\mathbf{r} = (x, y)$ and the integration over the imaginary time τ runs from zero to $\beta = 1/k_B T$. We work within the Weyl Gauge where we set the scalar potential $\phi(\mathbf{r}, t) = 0$. As the vector potential and current fluctuate around zero, we proceed by evaluating the free energy contribution arising from Eq. 28 within second-order perturbation theory. We present our results for the specific case where the electronic conductivity in the 2DES is local in space, finding the free energy [31]:

$$F_{\text{anis}} = T \sum_{\substack{\omega_m > 0 \\ a \in \{x,y\}}} \omega_m \sigma_{aa}(i\omega_m) \overline{\langle \mathbf{A}^a(i\omega_m) \mathbf{A}^a(-i\omega_m) \rangle}, \tag{29}$$

where the dynamical conductivity tensor, continued to imaginary Matsubara frequencies ($\omega_m = 2\pi m/\beta$), is given by $\sigma_{ij}(i\omega_m)$ and where the sample-averaged Matsubara frequency correlation function of the vector potential arising from the metamaterial cavity is given by $\overline{\langle \mathbf{A}^a(i\omega_m) \mathbf{A}^b(-i\omega_m) \rangle} = \int d^2\mathbf{R} \langle \mathbf{A}^a(\mathbf{R}, i\omega_m) \mathbf{A}^b(\mathbf{R}, -i\omega_m) \rangle$.

B. Stripe Anisotropy

Having articulated how to calculate the free energy arising due to light-matter coupling for a generic 2DES characterized by a local conductivity, we now turn towards computing the free energy for stripe configurations specifically. In particular, let us describe a particular stripe orientation by the angle θ formed by the wavevector $\hat{\mathbf{Q}}_{\text{stripe}}$ of the density modulation and the $\hat{\mathbf{y}}$-axis defined in Fig. 1C in the Main text (i.e. along the short axis of the cavity). For clarity, $\theta = 0$ ($\hat{\mathbf{Q}}_{\text{stripe}} \parallel \hat{\mathbf{y}}$) implies that the *easy axis* of transport is along the $\hat{\mathbf{x}}$-axis (hard axis of transport along the $\hat{\mathbf{y}}$-axis); $\theta = \pi/2$ ($\hat{\mathbf{Q}}_{\text{stripe}} \parallel \hat{\mathbf{x}}$) implies the reverse. Experimental findings are consistent with $\theta = 0$.

We now specify the dynamical conductivity of the stripes, generalizing the semi-classical Drude-like transport theory for the dc conductivity for stripes [32]—a theory that quantifies precisely the intuition of an "easy" and a "hard" direction—to finite (imaginary) frequencies: $\sigma_{ij}(i\omega_m) = \frac{1}{1+|\omega_m|\tau}\sigma_{ij}(\omega_m = 0)$, where $\sigma_{ii}(0) = \frac{e^2\sqrt{R}}{2h}$ and $\sigma_{jj}(0) = \frac{e^2}{2h\sqrt{R}}$; where the "easy" i axis is given by $\hat{\mathbf{e}}_i = \cos(\theta)\hat{\mathbf{e}}_x + \sin(\theta)\hat{\mathbf{e}}_y$ and the "hard" axis $j \perp i$; where the anisotropy ratio is $R = \frac{\sigma_{ii}}{\sigma_{jj}} \gg 1$. Here the relevant transport scattering time is $\tau = \frac{a\sqrt{R}}{v_F}$ [32], where v_F is the Fermi velocity and a is the inter-stripe separation (i.e. $|\hat{\mathbf{Q}}_{\text{stripe}}| = \frac{2\pi}{a}$). We note that the Drude transport within this model arises from disorder that gives rise to inter-stripe scattering, introducing both a finite conductivity for transport along the hard direction and a finite resistance to transport along the easy direction. It is precisely this disorder that allows $q = 0$ components of the electric field to couple to the conductivity of the electrons, eliding the restrictions imposed by Kohn's theorem. Leveraging this model for the dynamical conductivity—in the limit of $R \gg 1$—the stripe anisotropy per particle in the highest partially filled Landau level arising from vacuum fluctuations of the THz cavity is given by:

$$\Delta F_{\text{anis}} = \frac{Te^2\sqrt{R}}{2h} \times \sum_{\omega_m > 0} \frac{\omega_m}{1 + \omega_m \tau} (\overline{\langle \mathbf{A}^y(i\omega_m)\mathbf{A}^y(-i\omega_m)\rangle} - \overline{\langle \mathbf{A}^x(i\omega_m)\mathbf{A}^x(-i\omega_m)\rangle}) \cos(2\theta), \quad (30)$$

With a description of the electronic degrees of freedom in place, we articulate a model that captures the vacuum fluctuations of the slot antenna cavity, aiming to characterize $\overline{\langle \mathbf{A}^a(i\omega_m)\mathbf{A}^a(-i\omega_m)\rangle}$ through a decomposition in terms of the modes of the cavity. Neglecting dissipation,

$$\overline{\langle \mathbf{A}^a(i\omega_m)\mathbf{A}^a(-i\omega_m)\rangle} = \frac{1}{\epsilon_0\epsilon_r d} \sum_\lambda \frac{f^a_\lambda}{\omega_m^2 + \omega_\lambda^2}, \quad (31)$$

where λ runs over the modes of the cavity; where f^a_λ quantifies how much of the planar component of the mode λ is polarized along direction a (e.g. $f^x_\lambda = \frac{\int d^2R|A^x_\lambda(R)|^2}{\int d^2R|A^x_\lambda(R)|^2 + |A^y_\lambda(R)|^2}$); where $\epsilon_r = 13.1$ is the dielectric constant of GaAs; where the effective sub-wavelength confinement along the z-axis

is codified through $d \approx 100$ nm. An epistemically modest estimate of the anisotropy arises from considering the contributions from just the fundamental mode of the resonator, given that this is the only mode that has been unequivocally demonstrated to have strong coupling to electrons in the 2DES [24]. Here, as the mode is entirely polarized along the $\hat{\mathbf{y}}$-axis ($f^y_{\text{fund}} = 1, f^x_{\text{fund}} = 0$) it is clear that stripes will align with angle $\theta = 0$. Estimating this anisotropy leads to $\Delta F_{\text{anis}} \approx 100$ K ≈ 9 meV. It should be pointed out that the estimate, while sufficiently large to manipulate the orientation of the stripes, is not "large" as the value quoted is the *global* free-energy barrier for reorienting the stripes—which is what is relevant at the longest wavelengths. If one considers the *per particle* value—6 orders of magnitude smaller—the energy density remains safely within the perturbative regime.

REFERENCES

[1] J. Enkner, L. Graziotto, D. Boriçi, F. Appugliese, C. Reichl, G. Scalari, N. Regnault, W. Wegscheider, C. Ciuti, and J. Faist, Tunable vacuum-field control of fractional and integer quantum hall phases, Nature **641**, 884 (2025).

[2] M. Lilly, K. Cooper, J. Eisenstein, L. Pfeiffer, and K. West, Evidence for an anisotropic state of two-dimensional electrons in high landau levels, Physical Review Letters **82**, 394 (1999).

[3] R. Du, D. Tsui, H. Stormer, L. Pfeiffer, K. Baldwin, and K. West, Strongly anisotropic transport in higher two-dimensional landau levels, Solid State Communications **109**, 389 (1999).

[4] R. Ilan, N. R. Cooper, and A. Stern, Longitudinal resistance of a quantum hall system with a density gradient, Phys. Rev. B **73**, 235333 (2006).

[5] M. Buttiker, Symmetry of electrical conduction, IBM Journal of Research and Development **32**, 317 (1988).

[6] S. H. Simon, Comment on "evidence for an anisotropic state of two-dimensional electrons in high landau levels", Phys. Rev. Lett. **83**, 4223 (1999).

[7] J. Pollanen, K. Cooper, S. Brandsen, J. Eisenstein, L. Pfeiffer, and K. West, Heterostructure symmetry and the orientation of the quantum hall nematic phases, Physical Review B **92**, 115410 (2015).

[8] M. Fogler, A. Koulakov, and B. Shklovskii, Ground state of a two-dimensional electron liquid in a weak magnetic field, Physical Review B **54**, 1853 (1996).

[9] J. Zhu, W. Pan, H. Stormer, L. Pfeiffer, and K. West, Density-induced interchange of anisotropy axes at half-filled high landau levels, Physical Review Letters **88**, 116803 (2002).

[10] K. Cooper, J. Eisenstein, L. Pfeiffer, and K. West, Metastable resistance-anisotropy orientation of two-dimensional electrons in high landau levels, Physical Review Letters **92**, 026806 (2004).

[11] X. Fu, Q. Shi, M. Zudov, Y. Chung, K. Baldwin, L. Pfeiffer, and K. West, Quantum hall stripes in high-density gaas/algaas quantum wells, Physical Review B **98**, 205418 (2018).

[12] A. Dykhne and I. Ruzin, Theory of the fractional quantum hall effect: The two-phase model, Physical Review B **50**, 2369 (1994).

[13] F. von Oppen, B. I. Halperin, and A. Stern, Conductivity tensor of striped quantum hall phases, Physical Review Letters **84**, 2937 (2000).

[14] M. Sammon, X. Fu, Y. Huang, M. A. Zudov, B. I. Shklovskii, G. C. Gardner, J. D. Watson, M. J. Manfra, K. W. Baldwin, L. N. Pfeiffer, and K. W. West, Resistivity anisotropy of quantum hall stripe phases, Physical Review B **100**, 241303 (2019).

[15] M. Lilly, K. Cooper, J. Eisenstein, L. Pfeiffer, and K. West, Anisotropic states of two-dimensional electron systems in high landau levels: Effect of an in-plane magnetic field, Physical Review Letters **83**, 824 (1999).

[16] W. Pan, T. Jungwirth, H. Stormer, D. Tsui, A. MacDonald, S. Girvin, L. Smrcka, L. Pfeiffer, K. Baldwin, and K. West, Reorientation of anisotropy in a square well quantum hall sample, Physical Review Letters **85**, 3257 (2000).

[17] Q. Shi, M. Zudov, Q. Qian, J. Watson, and M. Manfra, Effect of density on quantum hall stripe orientation in tilted magnetic fields, Physical Review B **95**, 161303 (2017).

[18] D. Abanin, S. Morozov, L. Ponomarenko, R. Gorbachev, A. Mayorov, M. Katsnelson, K. Watanabe, T. Taniguchi, K. Novoselov, L. Levitov, and A. Geim, Giant nonlocality near the dirac point in graphene, Science **332**, 328 (2011).

[19] J. Wang and V. Goldman, Measurements and modeling of nonlocal resistance in the fractional quantum hall effect, Physical Review B **45**, 13479 (1992).

[20] P. McEuen, A. Szafer, C. Richter, B. Alphenaar, J. Jain, A. Stone, R. Wheeler, and R. Sacks, New resistivity for high-mobility quantum hall conductors, Physical Review Letters **64**, 2062 (1990).

[21] F. Appugliese, J. Enkner, G. L. Paravicini-Bagliani, M. Beck, C. Reichl, W. Wegscheider, G. Scalari, C. Ciuti, and J. Faist, Breakdown of topological protection by cavity vacuum fields in the integer quantum hall effect, Science **375**, 1030 (2022).

[22] A. Szafer, A. D. Stone, P. McEuen, and B. Alphenaar, Network models of the quantum hall effect, Granular Nanoelectronics , 195 (1991).

[23] M. Büttiker, Absence of backscattering in the quantum hall effect in multiprobe conductors, Physical Review B **38**, 9375 (1988).

[24] G. Scalari, C. Maissen, D. Turčinková, D. Hagenmüller, S. De Liberato, C. Ciuti, C. Reichl, D. Schuh, W. Wegscheider, M. Beck, and J. Faist, Ultrastrong coupling of the cyclotron transition of a 2d electron gas to a thz metamaterial, Science **335**, 1323 (2012).

[25] M. M. Fogler, Stripe and bubble phases in quantum hall systems, in *High Magnetic Fields: Applications in Condensed Matter Physics and Spectroscopy* (Springer, 2002) pp. 98–138.

[26] C. Ciuti, Cavity-mediated electron hopping in disordered quantum hall systems, Physical Review B **104**, 155307 (2021).

[27] R. Nicholas, R. Haug, K. v. Klitzing, and G. Weimann, Exchange enhancement of the spin splitting in

a gaas-ga x al 1- x as heterojunction, Physical Review B **37**, 1294 (1988).

[28] J. Matthews and M. Cage, Temperature dependence of the hall and longitudinal resistances in a quantum hall resistance standard, Journal of Research of the National Institute of Standards and Technology **110**, 497 (2005).

[29] J. Janak, g factor of the two-dimensional interacting electron gas, Physical Review **178**, 1416 (1969).

[30] A. Altland and B. Simons, *Condensed Matter Field Theory*, 3rd ed. (Cambridge University Press, Cambridge, UK, 2023).

[31] O. Carlsson, S. Chattopadhyay, J. B. Curtis, F. Lindel, L. Graziotto, J. Faist, and E. Demler, Casimir stabilization of fluctuating electronic nematic order, arXiv preprint arXiv:2510.05088 (2025).

[32] A. H. MacDonald and M. P. A. Fisher, Quantum theory of quantum Hall smectics, Physical Review B **61**, 5724 (2000).
